# Pravastatin reduces all-cause mortality in elderly individuals at risk of liver fibrosis: *Post hoc* analysis of the PROSPER trial

**DOI:** 10.1016/j.jhepr.2025.101337

**Published:** 2025-02-07

**Authors:** Vivian de Jong, Willy Theel, Manuel Castro Cabezas, Diederick E. Grobbee, Wouter Jukema, Stella Trompet

**Affiliations:** 1 Julius Center for Health Sciences and Primary Care, University Medical Center Utrecht, Utrecht, the Netherlands; 2 Julius Clinical, Zeist, the Netherlands; 3 Department of Internal Medicine, Franciscus Gasthuis & Vlietland, Rotterdam, the Netherlands; 4 Department of Endocrinology, Erasmus MC Medical Center, Rotterdam, the Netherlands; 5 Department of Cardiology, Leiden University Medical Centre, Leiden, the Netherlands; 6 Netherlands Heart Institute, Utrecht, the Netherlands; 7 Department of Internal Medicine, Section Gerontology and Geriatrics, Leiden University Medical Centre, Leiden, the Netherlands

**Keywords:** MASLD, FIB-4, Statin, Advanced fibrosis

## Abstract

**Background & Aims:**

Metabolic dysfunction-associated steatotic liver disease (MASLD), especially the progressive stages accompanied by liver fibrosis, are associated with liver-related and cardiovascular (CV) complications in middle-aged cohorts. We evaluated whether liver fibrosis is associated with increased mortality and cause-specific endpoints in an elderly population, and whether statin treatment could reduce these risks.

**Methods:**

PROspective Study of Pravastatin in the Elderly at Risk (PROSPER) was a double-blind randomized clinical trial comparing pravastatin to placebo in an elderly Caucasian population of 5,804 patients (>70 years of age) at increased risk of CV disease. Endpoints were composite and single (CV) endpoints and all-cause mortality. The Fibrosis-4 index (FIB-4) score was classified as: low risk of liver fibrosis (FIB-4 <2.0), indeterminate risk (2.0≤ FIB-4 ≤2.66), and high risk (FIB-4 ≥2.67). Time-to-event data were analyzed using the Cox proportional hazards model.

**Results:**

Most participants were classified in the low FIB-4 class (n = 3,919), followed by the indeterminate (n = 1,269) and high classes (n = 561). In the placebo group, the risk of all-cause mortality increased with a high FIB-4 classification: high-class hazard ratio (HR) = 1.54 (95% CI, 1.10–2.17), compared with the low class (reference group). In the pravastatin group, the HR for all-cause mortality was not associated with FIB-4 classification: high-class HR = 1.01 (95% CI, 0.69–1.49). The interaction between FIB-4 class and treatment was significant (*p* = 0.049). High FIB-4 classifications were not significantly associated with major adverse cardiovascular events (MACE) or other endpoints in either arms.

**Conclusions:**

A high FIB-4 classification is associated with increased all-cause mortality in the elderly, although pravastatin appears to mitigate this increased risk.

**Clinical Trials registration:**

The study is registered at www.isrctn.com/(ISRCTN40976937).

**Impact and implications:**

The progressive stages of MASLD (liver fibrosis) are associated with liver-related and CV complications in middle-aged cohorts (∼55 years of age). The impact of liver fibrosis in elderly populations is less well studied. In addition, the use of statins has long been debated, but evidence appears to point to a beneficial effect in populations with MASLD. However, data from prospective trials remain limited. Our findings indicate a potential survival benefit associated with pravastatin use in the elderly (>70 years of age) with an indication of liver fibrosis.

## Highlights

• The impact of liver fibrosis on the elderly (>70 years of age) is unclear.
• A high FIB-4 classification (≥2.67) was associated with increased all-cause mortality in people over 70 years of age.
• Pravastatin treatment lowered the risk of all-cause mortality in elderly participants with a high FIB-4 classification.

## Graphical abstract

**Figure:**
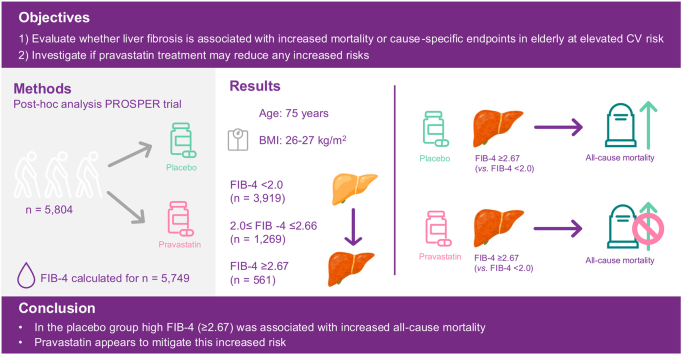

## Introduction

Metabolic dysfunction-associated steatotic liver disease (MASLD) can progresses from hepatic steatosis to liver fibrosis and inflammation, and may ultimately result in liver cirrhosis, decompensation, or hepatocellular carcinoma.^1^ Given the close association of MASLD with type 2 diabetes mellitus (T2DM) and obesity, its prevalence is also rapidly increasing in line with the increasing incidences of these conditions, with MASLD estimated to currently affect 38% of the global population.^2^ The pathogenesis of MASLD is multifactorial, with insulin resistance as a primary driver. In cohorts of middle-aged patients with biopsy-proven MASLD/metabolic dysfunction-associated steatohepatitis (MASH), the fibrosis stage is the most important predictor of adverse liver-related outcomes and mortality.[3], [4], [5] Whether liver fibrosis is also associated with mortality in older populations remains unclear.^6^

Lately, the cardiovascular (CV) implications of MASLD have received increased attention, as reflected in updated clinical guidelines^7^^,^^8^ and adoption of new nomenclature,^9^ wherein MASLD is defined as hepatic steatosis in the presence of cardiometabolic risk factors. MASLD has been linked to atherosclerotic CV disease (asCVD), heart failure, and an elevated risk of CV events in middle-aged patients.[10], [11], [12] The most common causes of death in patients with MASLD appear to be related to asCVD, rather than to the liver per se.^4^^,^^7^^,^^13^^,^^14^ This underscores the importance of addressing both hepatic and CV health in the management of patients with MASLD.

Recently, the first pharmacological treatment for MASLD was approved by the FDA.^15^ In Europe, the only available therapies are intensive lifestyle intervention and, in selected cases, bariatric surgery.^16^^,^^17^ However, the long-term success of lifestyle intervention remains low.^18^ Repurposing available drugs could provide immediate support and be more cost-effective. Hydroxymethylglutaryl-coenzyme A reductase inhibitors (statins) are a class of medication primarily used to lower LDL-cholesterol levels, thereby reducing asCVD risk. It has been suggested that statins could have additional anti-inflammatory and antifibrotic effects.[19], [20], [21], [22] The current study used the PROspective Study of Pravastatin in the Elderly at Risk (PROSPER) trial data^23^ to evaluate whether liver fibrosis is associated with increased mortality or cause-specific endpoints in older patients at elevated CV risk. We also investigated whether pravastatin treatment can reduce any increased risks.

## Patients and methods

### Study population

PROSPER was a randomized double-blind clinical trial investigating the effect of pravastatin in an older population with a history or increased risk of CVD (*i.e.* because of smoking, hypertension, or diabetes). The detailed study set-up has been published previously.^23^^,^^24^ Between 1997 and 1999, 5,804 people aged 70–82 years from Scotland, Ireland, and the Netherlands were included and randomized in a 1:1 ratio to receive either 40 mg pravastatin or matching placebo. The mean follow-up duration was 3.2 years and the study was completed in 2002.

### Endpoints

The composite endpoint of the trial was major adverse CV events (MACE), defined as definite or suspected coronary heart disease death, nonfatal myocardial infarction (MI), and either fatal or nonfatal stroke. The other endpoints included in this analysis were: fatal/nonfatal stroke and transient ischaemic attack (TIA); fatal/nonfatal MI; heart failure; all-cause mortality; cancer mortality; and CV mortality.

### FIB-4 score

The presence of liver fibrosis was estimated using the Fibrosis-4 Index (FIB-4)^25^ The FIB-4 algorithm is based on age, aspartate aminotransferase (AST), alanine aminotransferase (ALT), and platelet count, and was calculated at baseline using Equation 1:

[1] $$\text{FIB-}4=\left[\text{age}\;\left(\text{years}\right)\times \text{AST}\;\left(U/L\right)\right]/\left[\text{platelet}\;\text{count}\;\left(10^{9}/L)\times \sqrt{ALT}(U/L\right)\right]$$

Given that the population was above 65 years of age, we used the stricter age-corrected lower cut-off value (2.0 instead of 1.3) as proposed by McPherson *et al.*^26^ The population was split into three classes based on the following cut-off values: ruled-out advanced fibrosis (FIB-4 <2.0), indeterminate results (2.0 ≤ FIB-4 ≤2.66), and rule-in advanced fibrosis (FIB-4 ≥2.67). Reports from the LITMUS and LiverScreen consortia indicate that FIB-4 has adequate prognostic abilities at the population level.^27^^,^^28^

### Statistical analysis

Analyses were based on the intention-to-treat dataset. Population descriptive statistics were provided stratified by FIB-4 classification. Continuous data are presented as mean ± SE, and categorical data as count and proportion. The time-to-event data were analyzed with Cox proportional hazards models according to FIB-4 classification, for the placebo and pravastatin groups separately. For each endpoint, either time to ﬁrst occurrence of the event or study closure (censored observation) was taken, depending on which came first. The models were adjusted for sex, current smokers, BMI, diabetes, and history of CVD. For each endpoint, the hazard ratio (HR) and its 95% CI were estimated. Data analysis was conducted using SPSS (v. 29).

## Results

The baseline FIB-4 score was calculated for 5,749 participants of the total intention-to-treat population of 5,804 participants, given that the values used to calculate FIB-4 were missing for 55 participants (Fig. S1). The mean (±SE) FIB-4 in the placebo group and treatment group was similar (1.80 ± 0.01 placebo *vs.* 1.81 ± 0.01 treatment). Most participants were classified in the low FIB-4 class (n = 3,919), followed by the indeterminate class (n = 1,269), with fewest in the high FIB-4 class (n = 561). In the lowest FIB-4 class, most participants were female, whereas, in the indeterminate and high FIB-4 classes, most were male. The mean age (±SE) of the classes ranged from 74.9 (±0.07) to 76.4 (±0.21) years. Around 9–13% of the population had diabetes. A slightly increasing prevalence of a history of CVD was seen from the low (42%), to the indeterminate (47%) to the high FIB-4 (53%) classes (*p* <0.001). In terms of medication, use of metformin and insulin was minimal and did not differ between the classes. Use of angiotensin-converting enzyme (ACE)-/angiotensin II-inhibitors did not differ significantly between the classes or between the arms. The baseline characteristics are summarized in Table 1.

**Table 1:** Baseline characteristics of the PROSPER population stratified by FIB-4 classification and treatment.

	Placebo				Pravastatin			
	Low FIB-4	Indeterminate FIB-4	High FIB-4	*p* value	Low FIB-4	Indeterminate FIB-4	High FIB-4	*p* value
Participants (n)	1,975	636	273		1,944	633	288	
FIB-4	1.45 (0.01)	2.27 (0.01)	3.23 (0.04)	NA	1.45 (0.01)	2.28 (0.01)	3.18 (0.03)	NA
Age (years)	74.9 (0.07)	76.0 (0.13)	76.4 (0.21)	NA	75.1 (0.07)	75.9 (0.13)	76.0 (0.20)	NA
Females (n, %)	1,115 (56.5)	271 (42.6)	106 (38.8)	<0.001	1,088 (56.0)	286 (45.2)	105 (36.5)	<0.001
BMI (kg/m^2^)	26.9 (0.10)	26.7 (0.16)	26.2 (0.27)	0.007	27.0 (0.09)	26.6 (0.16)	26.1 (0.24)	<0.001
Current smokers (n, %)	581 (29.3)	146 (23.0)	70 (25.6)	0.010	540 (27.8)	151 (23.9)	58 (20.1)	0.002
Systolic blood pressure (mmHg)	154 (0.5)	156 (0.9)	156 (1.3)	0.081	154 (0.5)	155 (0.9)	156 (1.4)	0.203

**(Fasting) clinical values**								
Glucose (mmol/L)	5.48 (0.03)	5.44 (0.06)	5.49 (0.10)	0.771	5.49 (0.03)	5.35 (0.05)	5.39 (0.09)	0.061
Insulin (mIU/L)	10.6 (0.26)	9.6 (0.40)	9.8 (0.46)	0.070	10.5 (0.24)	10.1 (0.43)	10.5 (0.73)	0.660
HOMA-IR	2.64 (0.07)	2.45 (0.13)	2.53 (0.15)	0.262	2.64 (0.07)	2.47 (0.11)	2.59 (0.19)	0.409
Triglycerides (mmol/L)	1.57 (0.02)	1.50 (0.03)	1.35 (0.04)	<0.001	1.58 (0.02)	1.52 (0.03)	1.38 (0.04)	<0.001
HDL-cholesterol (mmol/L)	1.27 (0.01)	1.28 (0.01)	1.31 (0.02)	0.112	1.28 (0.01)	1.29 (0.02)	1.32 (0.02)	0.163
LDL-cholesterol (mmol/L)	3.82 (0.02)	3.73 (0.03)	3.63 (0.05)	<0.001	3.82 (0.02)	3.76 (0.03)	3.73 (0.05)	0.029
Creatinine (μmol/L)	100.3 (0.50)	102.8 (0.91)	101.3 (1.24)	0.067	101.0 (0.51)	102.1 (0.89)	102.5 (1.23)	0.165
AST (U/L)	22.9 (0.16)	27.9 (0.31)	33.4 (0.86)	NA	22.6 (0.16)	27.6 (0.33)	34.6 (0.80)	NA
ALT (U/L)	23.6 (0.24	23.3 (0.47)	23.7 (1.03)	NA	23.0 (0.22)	22.6 (0.45)	26.2 (1.08)	NA
Platelets (10^9^/L)	253.8 (1.22)	199.6 (1.41)	171.4 (2.02)	NA	254.4 (1.33)	199.1 (1.44)	170.0 (1.97)	NA

**Medical history**								
Diabetes (n, %)	217 (11.0)	64 (10.1)	34 (12.5)	0.792	210 (10.8)	56 (8.8)	31 (10.8)	0.501
Vascular disease (n, %)	811 (41.1)	291 (45.8)	146 (53.5)	<0.001	841 (43.3)	302 (47.7)	150 (52.1)	0.002

**Medication use**								
ACE inhibitors/angiotensin II inhibitors (n, %)	334 (16.9)	123 (19.3)	56 (20.5)	0.067	357 (18.4)	117 (18.5)	69 (24.0)	0.065
Metformin (n, %)	125 (6.3)	35 (5.5)	20 (7.3)	0.886	125 (6.4)	28 (4.4)	19 (6.6)	0.438
Insulin (n, %)	22 (1.1)	6 (0.9)	2 (0.7)	0.521	14 (0.7)	4 (0.6)	3 (1.0)	0.709

Continuous data are presented as mean (±SE), categorical data as count (%). FIB-4 classes: low FIB-4 <2.0; indeterminate FIB-4, 2.0–2.66; high FIB-4 ≥2.67. Numerical variables compared using ANOVA, categorical using Chi-square tests; *p* <0.05 were considered statistically significant (indicated in bold).

NA, testing not applicable, because these variables are part of the FIB-4 algorithm. ACE, angiotensin-converting enzyme; ALT, alanine aminotransferase; AST, aspartate aminotransferase; FIB-4, Fibrosis-4 index; HOMA-IR, Homeostatic model assessment for insulin resistance.

**Fig. 1:**
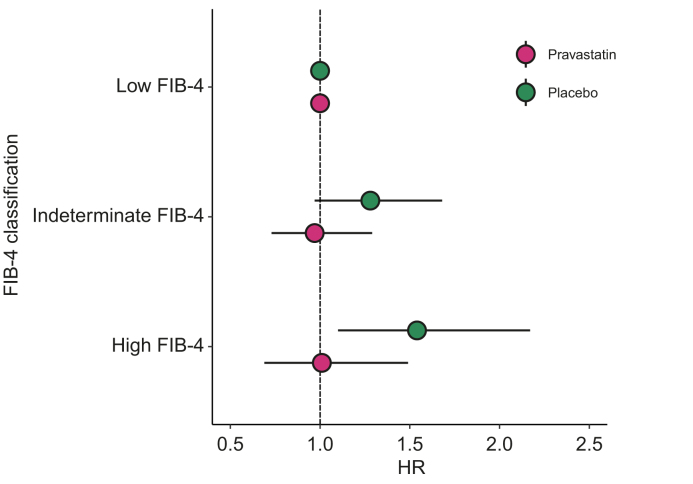
Hazard ratio (dots) and 95% CI (bars) for all-cause mortality for each FIB-4 classification compared to the reference class of low FIB-4 (HR set at 1) for the placebo and treatment group. The Cox proportional hazards model was adjusted for sex, smoking, BMI, diabetes, and history of vascular disease. ∗95% CI corresponding to alpha = 0.05, does not overlap with 1. FIB-4, Fibrosis-4 index; HR, hazard ratio.

A high FIB-4 classification was not significantly associated with the composite endpoint MACE (Table 2). In terms of the single endpoints, the risk of all-cause mortality was increased with higher FIB-4 classification in the placebo group (Fig. 1). In the placebo group, the high FIB-4 class had a HR of 1.54 (95% CI, 1.10–2.17) for all-cause mortality compared with the low FIB-4 class (reference group). However, in the pravastatin group, the HRs did not increase with a higher FIB-4 classification. In the pravastatin group, the high FIB-4 class had a HR of 1.01 (95% CI, 0.69–1.49) for all-cause mortality compared with the low FIB-4 class (reference group). The interaction term between FIB-4 class and statin treatment was significant (*p* = 0.049), indicating that the effect of treatment depended on FIB-4 classification (or vice versa). A higher FIB-4 classification was not associated with any of the other single endpoints studied: fatal/non-fatal stroke/TIA; fatal/nonfatal MI; heart failure; or cancer mortality (Table 2). A similar table showing only subjects that completed per protocol analysis is shown in Supplemental Material, Table S1.

**Table 2:** Hazard ratios and corresponding 95% CI for each endpoint for each FIB-4 class in the placebo arm and the treatment arm.

	Placebo (HR and 95% CI)			Pravastatin (HR and 95% CI)		
FIB-4 class	Low (n = 1,975)	Indeterminate (n = 636)	High (n = 273)	Low (n = 1,944)	Indeterminate (n = 633)	High (n = 288)
Major adverse cardiovascular events	1.0	0.98 (0.79–1.22)	0.82 (0.59–1.14)	1.0	1.03 (0.81–1.31)	0.98 (0.70–1.37)

**Single endpoints**						
Fatal/nonfatal stroke/transient ischemic attack	1.0	1.20 (0.79–1.81)	1.15 (0.65–2.04)	1.0	0.98 (0.64–1.52)	1.34 (0.79–2.26)
Fatal/nonfatal myocardial infarction	1.0	0.92 (0.71–1.19)	0.72 (0.48–1.06)	1.0	1.04 (0.78–1.38)	0.90 (0.60–1.36)
Heart failure	1.0	0.99 (0.64–1.52)	0.77 (0.40–1.49)	1.0	0.89 (0.55–1.44)	1.26 (0.71–2.24)
All-cause mortality	1.0	1.28 (0.97–1.68)	1.54 (1.10–2.17)∗	1.0	0.97 (0.73–1.29)	1.01 (0.69–1.49)
Cancer incidence	1.0	1.12 (0.80–1.57)	1.14 (0.72–1.82)	1.0	0.87 (0.63–1.20)	0.93 (0.60–1.43)
Cancer mortality	1.0	1.16 (0.70–1.91)	1.15 (0.57–2.33)	1.0	0.85 (0.53–1.36)	0.95 (0.50–1.79)
Cardiovascular mortality	1.0	1.39 (0.97–2.00)	1.11 (0.65–1.89)	1.0	1.08 (0.71–1.64)	1.15 (0.66–2.00)

Low FIB-4 is the reference class (HR set to 1). The Cox proportional hazards models were adjusted for sex, smoking, BMI, diabetes, and history of vascular disease. ∗95% CI corresponding to alpha = 0.05, does not overlap with 1.

FIB-4, Fibrosis-4 index; HR, hazard ratio.

## Discussion

In the current analysis of the PROSPER trial data, a high FIB-4 classification was associated with increased all-cause mortality in older patients in the placebo arm. However, pravastatin treatment appears to abolish this risk.

The most studied prognostic outcome in relation to FIB-4 is all-cause mortality. Systematic reviews and meta-analyses indicate that FIB-4 can accurately predict all-cause mortality.^27^^,^^29^ A individual participant data meta-analysis showed that FIB-4 is as good as histological fibrosis staging to predict a composite endpoint of all-cause mortality and liver-related outcomes (mean age 54 years).^30^ In our older population, a high FIB-4 classification (≥2.67) was also associated with an increased risk of all-cause mortality in the placebo arm. By contrast, an older general population cohort showed that neither liver steatosis nor liver fibrosis were associated with increased mortality (mean age 74 years, median follow-up almost 7 years).^31^ Whereas age and BMI were relatively similar between both studies, the prevalence of diabetes was higher in the general population cohort, whereas the presence of CVD was higher in our study. In addition, the assessment of fibrosis differed between the studies (FIB-4 *vs.* transient elastography). A registry-based study of Taiwanese patients with heart failure (mean age 75 years) also reported that FIB-4 was predictive of mortality in older people,^32^ although different FIB-4 cut-offs were used compared with the current analysis.

Several studies describe the prognostic relation between FIB-4 and CVD, although the results are less clear than for mortality. In a large meta-analysis, FIB-4 was not associated with CV events, although the number of events was relatively low (78 events in n = 2,518; 3%).^30^ By contrast, when using electronic health record data of patients with obesity and T2DM (6,002 CV events in n = 44,481; 13%) a higher FIB-4 classification was predictive of CV events (mean age 59 years).^33^ In the NHANES database, a high FIB-4 (≥2.67) was also predictive of CV events (mean age 53 years).^34^ In our older population, we did not find any association with MACE or any of the other CV endpoints. The power of our post hoc analysis might have been too low to detect differences in other single endpoints, although a relatively high number of combined CV events was observed for MACE (870 events in n = 5,749; 15%). However, the prospective relationships between FIB-4 and all-cause mortality might be stronger than for combined and single CV endpoints.

Statins are well known for their cholesterol-lowering effects. Lowering LDL-cholesterol might protect patients who are at increased risk of CV complications because of liver fibrosis, regardless of any potential effect on the fibrosis itself. Statins were associated with a decrease in mortality in patients with MASLD in the NHANES database.^22^ Our study confirms these retrospective NHANES findings, because we show that treatment with statins decreased all-cause mortality in PROSPER.

Statins might have additional anti-inflammatory and anti-fibrotic effects.^35^ Preclinical data demonstrated a beneficial effect of statins on diet-induced MASH^36^^,^^37^ and fibrosis.^37^ Only two small-sized randomized controlled trials on statins in MASLD have been performed,^38^^,^^39^ which were both classified as having a high risk of bias in the only Cochrane review to-date,^40^ and, therefore, no definite conclusion was reached. Given that no large-scale trials with adequate follow-up are available, the clinical evidence of statins in MASLD is limited to cohort data and post hoc analyses of RCTs. Statins were associated with protection against MASH and fibrosis in a cross-sectional study.^41^ Recently, two large meta-analyses reported that statin use was associated with a lower risk of MASH and fibrosis.^19^^,^^20^ Although statins are considered safe in patients with MASLD,^42^ older patients with liver fibrosis might be at a slightly increased risk of liver toxicity. In the current analysis, potential adverse effects did not compensate for the survival benefit. Nevertheless, in older populations, close monitoring of those with pre-existing liver conditions, multimorbidity, and polypharmacy remains important.

The prevalence of MASLD is higher in patients with T2DM compared with the general population,^43^ although this might be mainly driven by hepatic steatosis and insulin resistance. In PROSPER, the prevalence of T2DM was 9–13% and did not differ within the different fibrosis classifications.

To appreciate these findings, some aspects of this study need to be addressed. The strength of the current study is the use of a large dataset from a randomized controlled trial with an adequate follow-up time (4 years). The older study population at risk of CVD is comparable to patients who would be considered for statin treatment in practice. The main limitation of this study was the lack of direct liver assessment by either imaging or biopsy, and FIB-4 was selected as proxy for liver fibrosis. FIB-4 has been validated as a diagnostic marker for advanced fibrosis at the population level, as reflected in available guidelines.^44^ However, the diagnostic accuracy of FIB-4 in specific subpopulations, such as patients with T2DM, is lower. The accuracy of its use as prognostic marker is also less studied, although recent data show that FIB-4 has discriminative power in patients with T2DM.^33^ In addition, FIB-4 is mostly validated (as a diagnostic marker) in populations under 65 years of age. The FIB-4 algorithm includes age and, thus, likely overestimates the presence of fibrosis in older participants. Therefore, in this analysis, we used age-specific cut-offs as proposed by McPherson *et al.*^26^ and adopted in the 2024 EASL–EASD–EASO Clinical Practice Guidelines.^8^ PROSPER compared pravastatin with placebo, while other types of statin (i.e., simvastatin and atorvastatin) are more commonly prescribed. Given that the mechanism of action of statins is similar, we expect the results to also be generalizable to other statins. A high level of high intraindividual variation was observed in this study, possibly because the response to statin treatment might differ based on underlying genotype or metabolic profile.^41^

Non-randomized studies have indicated potential benefits of statin use in middle-aged patients with MASLD,^19^^,^^22^^,^^41^ but no consensus or standard recommendation has been reached. Our analysis of PROSPER suggests that the beneficial effects of statins also extend to an older population with a high risk of liver fibrosis.

In conclusion, our findings obtained in a large population of older participants at elevated CV risk support the view that increased risks of fibrosis lead to increased risks of all-cause mortality. Moreover, the results of a randomized comparison suggest that pravastatin treatment, or possibly statin treatment in general, mitigates these risks.

## Abbreviations

ACE, angiotensin-converting enzyme; ALT, alanine aminotransferase; asCVD, atherosclerotic CVD; AST, aspartate aminotransferase; CV, cardiovascular; CVD, cardiovascular disease; FIB-4, Fibrosis-4; HOMA-IR, Homeostatic Model Assessment for Insulin Resistance; HR, hazard ratio; MACE, major adverse cardiovascular events; MASH, metabolic dysfunction-associated steatohepatitis; MASLD, Metabolic dysfunction-associated steatotic liver disease; MI, myocardial infarction; PROSPER, PROspective Study of Pravastatin in the Elderly at Risk; T2DM, type 2 diabetes mellitus; TIA, transient ischemic attack.

## Financial support

The authors received no specific funding for this work. The original PROSPER study was supported by an investigator-initiated grant obtained from Bristol-Myers Squibb, USA. Prof. Dr. J. W. Jukema is an Established Clinical Investigator of the Netherlands Heart Foundation (grant 2001 D 032). The funders had no role in study design, data collection and analysis, decision to publish, or preparation of the manuscript.

## Authors’ contributions

Study concept and design; MCC, ST, VDJ. Statistical analysis; ST. Interpretation of data; ST, VDJ, MCC, DG. Drafting of the manuscript; VDJ. Critical revision of the manuscript; MCC, ST, DG, WT, WJ. Supervision: MCC, DG.

## Data availability statement

Data cannot be shared publicly due to ethical constraints, but can be requested from the PROSPER Scientific Committee (vice chairman of the PROSPER Scientific Committee: Naveed Sattar, naveed.sattar@glasgow.ac.uk) or from the co-author and PROSPER PI J.W. Jukema (j.w.jukema@lumc.nl).

## Conflicts of interest

The authors have declared no competing interests.

Please refer to the accompanying ICMJE disclosure forms for further details.
