# Supplementary material for: Pravastatin reduces all-cause mortality in elderly individuals at risk of liver fibrosis: Post hoc analysis of the PROSPER trial
Source: JHEP Rep. 2025 Feb 7;7(4):101337. doi: 10.1016/j.jhepr.2025.101337 (PMC11985108; doi:10.1016/j.jhepr.2025.101337)
Supplement: Multimedia component 1 [file mmc1.pdf]

Pravastatin reduces all-cause mortality in elderly individuals at risk of liver fibrosis: *Post hoc* analysis of the PROSPER trial

Vivian Desiree de Jong, Willy Theel, Manuel Castro Cabezas, Diederick Grobbee, Wouter Jukema, Stella Trompet

Table of contents

Fig. S1…………………………………………………………………………………….2

Table S1…………………………………………………………………………………..3

Fig. S1: patient flow through the original trial, with addition of missing FIB-4. **The** ITT population was n=2,913 for placebo and n=2,891 for treatment. In the placebo for n=29 values to calculate FIB-4 were missing, in the treatment group for n=26 values to calculate FIB-4 were missing. Resulting in a total population of n=2,884 in placebo and n=2,865 for treatment for this post-hoc analysis.

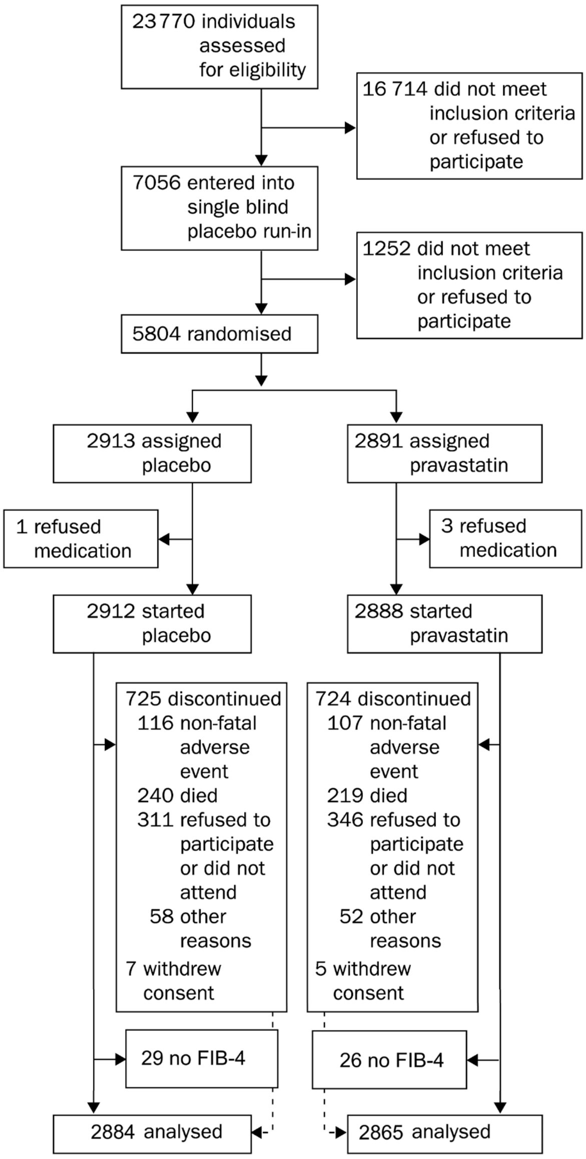

Table S1: Hazard ratios (HR) and corresponding 95% CI for each endpoint for each FIB-4 class in the placebo arm and the treatment arm. Low FIB-4 is the reference class (HR set to 1). The Cox proportional hazards models were adjusted for sex, smoking, BMI, diabetes and history of vascular disease. ONLY in subjects that completed the per protocol analysis (people that withdraw consent were left out). *95% confidence interval corresponding to alpha=0.05, does not overlap with 1.

	Placebo			Pravastatin		
**FIB-4 class**	Low N=1681	Indeterminate N=549	High N=244	Low N=1640	Indeterminate N=553	High N=253
**MACE**	1.0	0.98 (0.77-1.26)	0.89 (0.63-1.26)	1.0	1.00 (0.77-1.30)	0.97 (0.67-1.40)
**Single endpoints**						
**Fatal/non-fatal Stroke/TIA**	1.0	1.28 (0.79-2.05)	1.42 (0.77-2.60)	1.0	0.81 (0.48-1.37)	1.34 (0.75-2.40)
**Fatal/nonfatal MI**	1.0	0.90 (0.68-1.19)	0.76 (0.50-1.14)	1.0	1.07 (0.79-1.44)	0.91 (0.59-1.41)
**Heart failure**	1.0	0.88 (0.54-1.44)	0.80 (0.40-1.61)	1.0	0.96 (0.57-1.61)	1.42 (0.77-2.61)
**All-cause mortality**	1.0	1.26 (0.96-1.65)	1.47 **(1.05-2.07)***	1.0	0.94 (0.71-1.25)	0.97 (0.66-1.43)
**Cancer Incidence**	1.0	1.24 (0.87-1.77)	1.19 (0.73-1.94)	1.0	0.83 (0.58-1.18)	0.94 (0.59-1.49)
**Cancer mortality**	1.0	1.31 (0.69-1.87)	1.08 (0.54-2.19)	1.0	0.81 (0.51-1.31)	0.91 (0.48-1.72)
**CV mortality**	1.0	1.38 (0.96-1.98)	1.07 (0.63-1.82)	1.0	1.38 (0.96-1.98)	1.07 (0.63-1.98)
