# Supplementary material for: Pravastatin reduces all-cause mortality in elderly individuals at risk of liver fibrosis: Post hoc analysis of the PROSPER trial
Source: JHEP Rep. 2025 Feb 7;7(4):101337. doi: 10.1016/j.jhepr.2025.101337 (PMC11985108; doi:10.1016/j.jhepr.2025.101337)
Supplement: Multimedia component 4 [file mmc4.zip › Clinical trials/PROSPER protocol_AM J Cardiol 1999.pdf]

The Design of a Prospective Study of *Pravastatin* in the Elderly at Risk (PROSPER)

James Shepherd, MD, Gerard Jan Blauw, MD, Michael B. Murphy, MD, Stuart M. Cobbe, MD, Edward L. E. M. Bollen, MD, Brendan M. Buckley, MD, Ian Ford, PhD, J. Wouter Jukema, MD, Michael Hyland, MD, Allan Gaw, MD, A. Margot Lagaay, MD, Ivan J. Perry, MD, Peter W. Macfarlane, PhD, A. Edo Meinders, MD, Brian J. Sweeney, MD, Chris J. Packard, DSc, Rudi G. J. Westendorp, MD, Cillian Twomey, MD, and David J. Stott, MD, on behalf of the PROSPER Study Group

The PROspective Study of Pravastatin in the Elderly at Risk (PROSPER) is a randomized, double-blind, placebo-controlled trial designed to test the hypothesis that treatment with pravastatin will diminish risk of subsequent major vascular events in a cohort of men and women (70 to 82 years old) with preexisting vascular disease or significant risk of developing this condition. Five thousand eight hundred four men and women in addition to receiving advice on diet and smoking, have been randomized equally to treatment with 40 mg pravastatin/day or matching placebo in 3 centers (Cork, Ireland, Glasgow, Scotland, and Leiden, The Netherlands). Following an average 3.5-year intervention period, a primary assessment will be made of the influence of this therapy on major vascular events (a combination of coronary heart disease, death, nonfatal myocardial infarction, and fatal and nonfatal stroke). A number of additional analyses will also be conducted on the individual components of the primary end point, on men, on women, and on subjects with and without previous evidence of vascular disease. Finally, an assessment will be made of the effects of treatment on cognitive function, disability, hospitalization or institutionalization, vascular mortality, and all-cause mortality. **©1999 by Excerpta Medica, Inc.**

(Am J Cardiol 1999;84:1192–1197)

There is growing evidence that statins may reduce the risk of vascular disease in subjects >70 years of age, just as they do in younger individuals. However, this has not been subjected to formal scrutiny in a dedicated large scale trial targeting clinical end points. In the elderly, arterial disease remains the leading cause of death and disability[1,2] and is considered to be a major contributor to cognitive decline and dementia.[3] Strategies aimed at reducing the physical and mental impairment associated with aging may decrease end-of-life morbidity, improve active life expectancy, and, in consequence, yield substantial economic benefits from reduced hospitalization and need for nursing home care.[4,5] The present study aims to determine whether treatment with pravastatin is likely to influence this process.

STUDY DESIGN

The PROspective Study of Pravastatin in the Elderly at Risk (PROSPER) is a double-blind, randomized, placebo-controlled trial designed to examine the hypothesis that pravastatin, at a dose of 40 mg/day, will reduce the risk of cardiovascular and cerebrovascular events in elderly subjects with vascular disease or at high risk of developing vascular disease. Three European coordinating centers—Glasgow, Scotland, Cork, Ireland, and Leiden, The Netherlands—are collaborating in the project, which is based in primary care (general practice) or in trial centers in close proximity to each of the 3 coordinating centers.

Study subjects and recruitment policy: Five thousand eight hundred four elderly men and women (2,806 men, 2,998 women), 70 to 82 years of age, with plasma total cholesterol of 4.0 to 9.0 mmol/L (155 to 350 mg/dl), triglycerides ≤6.0 mmol/L (530 mg/dl), and good cognitive function (Mini Mental Score ≥24 of 30 at baseline[6]), have been enrolled into this study, using the eligibility criteria outlined in Table I. Approximately 50% of the study population have evidence of vascular disease, and the other 50% are at high risk for vascular disease because they have ≥1 major vascular risk factors (hypertension, cigarette smoking, or diabetes mellitus).

Individuals were identified in the primary care setting during a 10-week screening and enrollment program which, for eligible candidates, will be followed by a double-blind treatment period. After 2 initial screening visits conducted by a study nurse, eligible subjects entered a 4-week single-blind pla-

From the University of Glasgow, Glasgow, Scotland; the University of Leiden, Leiden, The Netherlands; and the University of Cork, Cork, Ireland. This study is supported by a research grant from the Bristol-Myers Squibb Pharmaceutical Research Institute, Princeton, New Jersey. Manuscript received February 12, 1999; revised manuscript received and accepted June 16, 1999.

Address for reprints: James Shepherd, MD, Department of Pathological Biochemistry, Royal Infirmary, Glasgow G4 OSF, Scotland. E-mail: JS4N@clinmed.gla.ac.uk.

 ©1999 by Excerpta Medica, Inc. All rights reserved.

0002-9149/99/$–see front matter
PII S0002-9149(99)00533-0

TABLE I Inclusion and Exclusion Criteria

Inclusion criteria
Men or women aged 70–82 yrs
Total cholesterol 4.0–9.0 mmol/L
Physician diagnosed stable angina or intermittent claudication
Stroke, transient ischemic attack, myocardial infarction, arterial surgery, or amputation for vascular disease >6 months before study entry
≥1 of the following risk factors for vascular disease
Current smoker
Hypertension, currently receiving drug treatment
Known diabetes mellitus or fasting blood glucose >7 mmol/L
Exclusion criteria
Recent stroke, transient ischemic attack, myocardial infarction, arterial surgery, or amputation for vascular disease ≤6 months before study entry
Any surgery requiring overnight hospitalization for a medical reason (including angioplasty) ≤6 months before study entry
Poor cognitive function at baseline (Mini Mental Score Examination <24)
Physically or mentally unable to attend the clinic for the screening visit
Total cholesterol <4.0 mmol/L or total cholesterol >9.0 mmol/L
Total triglycerides >6.0 mmol/L
History of malignancy within the past 5 years except localized basal cell carcinoma of the skin
Congestive heart failure (New York Heart Association functional class III or IV)
Electrocardiographic evidence of atrial fibrillation or other significant arrhythmia, or Wolff-Parkinson-White syndrome
Implanted cardiac pacemakers with the capacity for ventricular pacing
Organ transplant recipient
Current lipid-lowering drug treatment
Cyclosporin treatment
Previous participation in a clinical trial using an HMG CoA reductase inhibitor
Inability to give informed consent
Planned long-term travel or emigration within next 3 years
Current alcohol or drug abuse
Cohabitation with another trial participant
<75% or >120% compliance with placebo lead-in medication
Receipt of any investigational drugs (including placebo) within 30 days of enrollment
Inability to tolerate oral medication or a history of significant malabsorption
Any other medical condition that renders the patient unable to complete the study or that would interfere with optimal participation in the study or produce significant risk to the patient
Abnormal laboratory findings, including
Hemoglobin <11 g/dl and hematocrit <33%
Thyroid stimulating hormone >20 U/L or >10 U/L with an abnormal free thyroxine
Glucose >15 mmol/L
Platelet count <100,000/mm^3
White blood cell count <3,500/mm^3 or >15,000/mm^3
Serum creatinine >200 μmol/L
Aspartate aminotransferase or alanine aminotransferase >3.0 × upper limit of normal for the laboratory
Creatine kinase >3 × upper limit of normal for the laboratory

cebo lead-in period (Figure 1). At the end of the placebo lead-in, the 5,804 subjects who continued to satisfy the enrollment criteria (as assessed by a physician) and were ≥75% compliant with placebo medication (by tablet count) were randomized on a double-blind basis and in a 1:1 ratio to receive pravastatin 40 mg or placebo once daily for a minimum of 3 years to an expected maximum of 5 years (Figure 1).

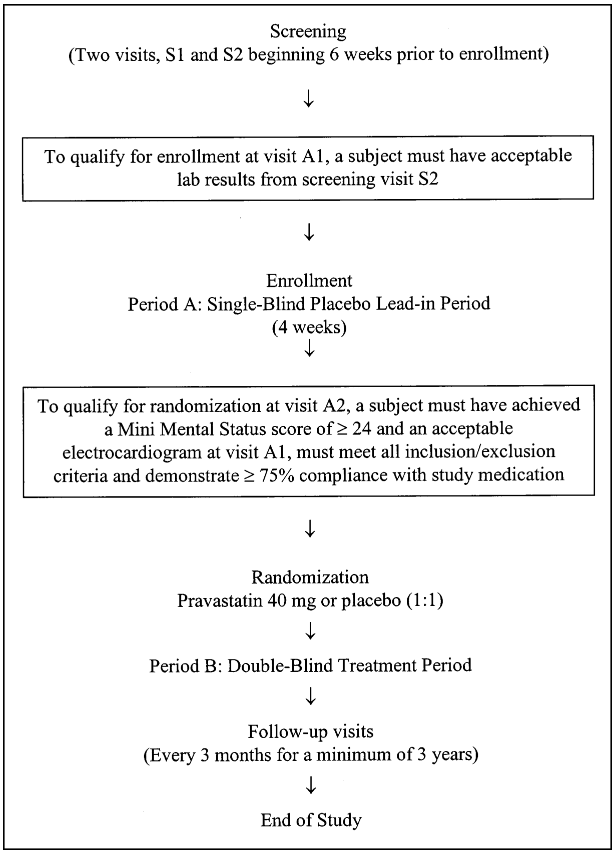

FIGURE 1. Flowchart of study.

Throughout the project, all subjects will receive nutritional advice and health counseling, and will be exhorted to follow the National Cholesterol Education Program Step 1 diet or a local equivalent that will provide <30% of total calories from fat (<10% as saturated fat) and a cholesterol intake of <300 mg/day. During the double-blind phase, subjects will be seen at clinic visits in the trial centers every 3 months. Integral to the project are a number of ongoing assessments necessary for an analysis of resource utilization and cost benefit that will be performed as a substudy.

Study end points: The primary outcome measure is the combined end point of coronary heart disease death (definite plus suspect), definite plus suspect nonfatal myocardial infarction, and fatal plus nonfatal stroke.

Secondary analyses will be performed for the primary end point in men, women, and subjects with previous vascular disease in the form of stable angina, intermittent claudication, stroke, transient ischemic attacks, myocardial infarction, arterial surgery, or amputation for vascular disease >6 months before study entry, and in individuals without evidence of previous vascular disease but who are considered to be at high risk on the basis of being a current smoker, diabetic, or hypertensive. Central to the project is an assessment of whether pravastatin will successfully retard cognitive decline and disability and obviate the need for long-term hospitalization or institutionalization.

For the sake of brevity, secondary and tertiary

outcome measures and detailed end point definitions are presented in Appendix B.

Concomitant medication: Prescription of nonstudy medication is expected to be a frequent occurrence in this trial. Consequently most drugs, with the exception of lipid-lowering agents (other than the study medication) and cyclosporin, are permitted during the project. In particular, postmenopausal women receiving stable estrogen replacement therapy or individuals who are euthyroid on stable doses of thyroid hormones may continue use of these agents during the trial. Low-dose aspirin therapy ($\leq$325 mg/day), the use of topical steroids, or steroid nasal sprays and stable customary caffeine intake is permissible.

If during the course of the trial a participant's personal physician considers it necessary for some reason to initiate unblinded lipid-lowering drug therapy, the following procedures will be recommended. In the first instance, the physician will be encouraged to use pravastatin and to continue to allow the participant to take the trial medication. Should the participant be on active pravastatin within the trial, the total daily dose which the patients would then be receiving would be 40 mg plus the treatment dose selected by the physician. The maximum dose prescribed would therefore be 80 mg/day. There are clinical data that support the safety of this dose of pravastatin.[7,8] Maintaining the patient on randomized therapy by this approach will help retain the difference in cholesterol between active and placebo groups, even though additional lipid-lowering therapy is administered. Should the patient's physician find this unacceptable, and prefer to prescribe a cholesterol lowering drug other than pravastatin, the recommendation is that study medication is discontinued as the safety of other lipid-lowering drugs combined with pravastatin is not known in this age group. The subject, however, will continue to be followed within the regular visit schedule of the project to safeguard its "intention-to-treat" design status.

Safety issues: Timely and complete reporting of all adverse events will permit a greater understanding of the overall safety profile of the drug (particularly in this age range of subjects who are likely to be receiving a number of other pharmaceutic agents), will facilitate identification of dose-related drug toxicity, and will accelerate appropriate modification of study protocols. Any illness, sign, symptom, or clinically significant laboratory test abnormality that has appeared or worsened during the course of the clinical trial will be pursued regardless of the causal relation to the active pravastatin therapy. If known, the underlying diagnosis will also be recorded, rather than its individual symptoms.

Subjects experiencing adverse events that are present at the end of their participation in the study will receive follow-up as appropriate. Strenuous efforts will be made to determine the outcome of any adverse event that caused permanent discontinuation or that was present at the end of the study, particularly if the adverse event was considered by the investigator to be probably and/or possibly related, or of undetermined relation to the study drug. Serious adverse events, defined as fatal or life threatening, permanently disabling, or requiring or prolonging inpatient hospitalization, will be handled expeditiously according to regulatory requirements. Cancer and drug overdosage will also be handled as serious adverse events.

STUDY VISITS

Prerandomization visits: At their initial visit (see Figure 1) individuals gave their informed consent to be screened. A brief medical history was taken, vital signs recorded, and appropriate health and dietary advice given. If the subject satisfied the eligibility criteria determined at this visit, the patient was invited to attend the second screening visit. At this visit (S2, Figure 1), a more detailed medical history was taken, a fasting venous blood sample collected for biochemical and hematologic checks and for lipoprotein (beta) quantification, and concomitant medication rechecked.

Fasting subjects were then invited to attend the first enrollment visit (A1, Figure 1) if on the basis of the results of their blood tests at S2 they qualified to continue the study. There, a second fasting venous blood sample was drawn for repeat lipid and lipoprotein profiling and a sample stored in the PROSPER bio-bank. In addition, a Mini-Mental State Examination,[6] the Picture-Word Learning Test,[9] the Stroop Colour Word Test,[10,11] and the Letter Digit Coding Test[12,13] were conducted. The Activities of Daily Living (modified 20-point Barthel index)[14] and Instrumental Activities of Daily Living[15] questionnaires were also administered. A 12-lead electrocardiogram was recorded.

Unless any obvious exclusion was encountered in the Mini-Mental State Examination or the electrocardiogram, the subject was issued placebo run-in medication on a single-blind basis.

The final enrollment visit (A2, Figure 1) culminated in randomization of participants into the study. If compliance to the placebo run-in medication was 75% to 120% of the expected value, the subject's weight, height, and blood pressure were recorded and the mental and physical ability tests readministered. The study investigator then conducted a medical examination and made a final check of the study inclusion and exclusion criteria (Table I). If all criteria were met, the subject's consent to the study was endorsed by the patient's general practitioner as a study subinvestigator. The PROSPER Data Center was then contacted for a randomization number, and study medication issued in a double-blind fashion. Randomization was performed using the method of permuted blocks, stratified by site.

Within-trial visits: Three months after randomization, compliance with study medication will be rechecked by pill counting. Subjects will be counseled on healthy eating and details of any adverse events recorded. Concomitant medication will be noted, and the next pack of study medication dispensed. Blood pressure and weight will be recorded and a fasting venous blood sample drawn for lipid and lipoprotein

TABLE II Power Calculations for PROSPER

Subgroup	Event	No. per Group	Placebo Rate	Percent Reduction	Power
All subjects	All CV events	2,750	16%	20%	92%
All subjects	Stroke	2,750	8%	28%	90%
All subjects	CHD events	2,750	12%	25%	95%
Men	All CV events	1,250	19%	25%	89%
Women	All CV events	1,500	14%	25%	83%
Previous vascular disease	All CV events	1,375	22.5%	20%	83%
No previous vascular disease	All CV events	1,375	10%	30%	80%

CHD = coronary heart disease; CV = cardiovascular.

profiling, biochemistry and hematology safety checks, and for storage in the PROSPER bio-bank. At 6 months, the lipid and lipoprotein profile will be measured, vital signs recorded, and concomitant medication noted. Compliance with study medication will be checked as before and the next pack of study medication dispensed.

The 9-month visit will incorporate a recheck of cognitive function; vital signs will also be recorded and concomitant medication noted. Following a compliance check, the next pack of study medication will be dispensed and any adverse events identified. After 1 year, a fasting venous blood sample will be drawn for lipid and lipoprotein profiling, a 12-lead electrocardiogram performed, and new study medication issued.

Thereafter, study visits will be conducted approximately every 3 months. At yearly intervals, repeat tests of lipid and lipoprotein profiles, 12-lead electrocardiogram, and cognitive function will be performed.

Cognitive function tests: The Mini Mental State Examination (MMSE)[6] is widely used to screen for cognitive dysfunction. In the present study the generally used cut-off criterion of 24 points will be used. Individuals scoring below that level at screening will be excluded.

Subjects' performance of 3 cognitive tests will serve as outcome variables. Verbal learning will be assessed using the Picture-Word Learning Test derived from the Groningen Fifteen Words Test,[9,16] originally described by Rey.[17]

The Letter-Digit Coding Test is a modification of the procedurally identical Symbol-Digits Modalities Test. This test is used to measure the speed of processing of general information and draws upon several cognitive processes simultaneously, such as visual scanning, perception, visual memory, visuoconstruction, and motor functions.

The Stroop Color-Word Test has often been used to measure selective attention.[10] It comprises 3 parts, each containing 100 elements: color names, colored patches, and color names printed in incongruously colored ink. The time required to read the names or to identify colors is recorded. For the purposes of the present study, an abbreviated version will be used[11] in which the stimuli are reduced to 40 items per element of the test.

Sample size and statistical considerations: SAMPLE SIZE: The study was designed to have adequate power to show significant reductions in each of the primary and secondary outcomes and for the primary outcome in the subgroups of men, women, and those with and without previous evidence of vascular disease. All power calculations were based on the use of the log-rank test with a significance level of 5%, to compare time-to-event outcomes between treatment groups. It was initially assumed that the study population will consist of 3,000 women and 2,500 men and that there would be 2,750 subjects with a history of vascular disease and 2,750 subjects with no history of vascular disease, but at high risk on the basis of smoking history, diabetes, or hypertension. The statistical power associated, given percent risk reductions from assumed placebo risks with an average of 3.5 years of follow-up, is shown in Table II. It is estimated that a total of 792 cardiovascular events will be required to obtain 92% power for the primary end point. A significant difference of $p < 0.001$ (Peto type rule) for all-cause mortality will be used as a guideline for recommending early discontinuation of the trial. However, highly significant results for other major end points will also be taken into consideration.

Statistical analysis: The primary method of analysis for all primary and secondary end points will be the Cox proportional hazards model. The Cox proportional hazards model for all outcomes will be adjusted for the following major baseline risk factors: smoking status (current smoker or not), blood pressure, antihypertensive treatment (yes or no), age, sex, diabetes (yes or no), history of vascular disease (yes or no), electrocardiographic abnormalities (evidence of myocardial infarction, ST-T wave changes, left ventricular hypertrophy, conduction defects), and baseline low- and high-density lipoprotein cholesterol levels. The outcome measurement for each variable will be the time to first occurrence of the event or study closure (censored observation), whichever comes first. The primary analysis will be based on the intention-to-treat principle. The proportional hazards assumption will be checked for each end point. In addition, the hazard ratio for the treatment effect will be estimated along with its 95% confidence interval. The relation between the magnitude of treatment effect and levels of baseline risk factors will be investigated within the Cox proportional hazards model. The relation between on-treatment lipid levels and outcome, in the group randomized to pravastatin, will be investigated using a time-dependent Cox proportional hazards model.

Other analyses: Blood cell samples from each participant are being stored with a view to undertaking analyses of gene variations, which may be linked to vascular disease risk.

ORGANIZATIONAL STRUCTURE

Administrative centers: Three national administrative centers, based in each of the participating Universities are responsible for recruiting the study centers, providing clinician support to the study nurses and subinvestigators, managing the flow of trial medication and data, recording and reporting adverse events, and servicing the study committees.

Data center: The data center, located at the university of Glasgow in the Robertson Center for Biostatistics is responsible for the central randomization system, case report form design, data entry and validation, and providing blinded reports to the Executive Committee and unblinded reports to the Data and Safety Monitoring Committee. The data center will be also responsible for all statistical analyses leading to scientific publications.

Field operations: Minerva Medical plc (Glasgow, Scotland), a subject recruitment company, provides, trains, and organizes the study nurses who conduct all the study visits according to the Protocol and Manual of Operations.

Central lipid laboratory: The Department of Pathological Biochemistry at the Glasgow Royal Infirmary is the central laboratory for all lipid analyses. The central laboratory participates in the standardization program of the Centers for Disease Control in Atlanta, Georgia. See the PROSPER Website for complete details (http://www.gla.ac.uk/Acad/PathBio/prosper.htm).

Electrocardiographic core laboratory: The University of Glasgow Department of Medical Cardiology based at the Glasgow Royal Infirmary, Scotland, is the Core Laboratory for all electrocardiographic analyses. All recordings made throughout the study are transmitted there electronically for storage and automated Minnesota Coding.

Study centers: All study visits will be conducted by a dedicated PROSPER Study nurse in the office of the subject's own general practitioner (Ireland and Scotland) or in a trial center nearby (The Netherlands).

Bio-bank: The central bio-bank for the secure storage of blood, plasma, serum, and cells is located in Leiden University Medical Center.

MANAGEMENT STRUCTURE AND COMMITTEES

All members of the committees are listed in Appendix A.

Several committees will be responsible for managing the study and monitoring the safety of the study subjects. The Executive Committee will serve as the primary management group for the study and will be responsible for formulating policy and directing the activities of the other committees.

The Data and Safety Monitoring Committee will serve as an external advisory group to the Executive Committee and be responsible for reviewing unblinded safety data for the trial at scheduled intervals. In addition, the Committee will review efficacy data on an annual basis and be responsible for taking appropriate action if the data suggest that there may be an unacceptable risk to the subjects in the study.

The Endpoints Committee will be responsible for the classification of all possible study end points. The Committee will receive all annual study electrocardiograms showing serial changes, information regarding domiciliary visits or hospitalizations associated with possible cardiovascular events, and information on all deaths (including postmortem reports and/or certification of death).

Finally, the Publications Committee will include all voting Executive and Endpoint Committee members, and the project statistician. The Committee will have responsibility for all study-related publications.

DISCUSSION

Coronary artery disease is the prime cause of death in older people, and stroke, often accompanied by vascular dementia, ranks highest as a source of disability. The fact that treatment of middle-aged individuals with pravastatin[18,19] reduces their risk of heart attack and stroke augurs well for the elderly. However, it is inappropriate to extrapolate the stroke reductions seen in middle-aged subjects who have a history of myocardial infarction to the broader sweep of elderly individuals at risk of a cerebrovascular event.[20,21] The need to demonstrate and quantify this benefit provides the rationale for PROSPER.

APPENDIX A

Executive Committee: *Cork, Ireland*: Professor Michael B Murphy (Principal Investigator); Dr. Brendan M Buckley (Pharmacology and Therapeutics); Professor Ivan J. Perry (Epidemiology and Public Health); Dr. Michael Hyland (Geriatrics). *Glasgow, Scotland*: Professor Jim Shepherd (Chairman/Principal Investigator); Professor Chris J. Packard (Study Director); Dr. Allan Gaw (Deputy Study Director); Professor Stuart M. Cobbe (Cardiology); Professor David J. Stott (Geriatrics). *Leiden, The Netherlands*: Dr. Gerard J. Blauw (Principal Investigator); Dr. Rudi G. J. Westendorp (Gerontology and Geriatrics); Dr. A. Margot Lagaay (Gerontology and Geriatrics); Dr. Edward L. E. M. Bollen (Neurology). *Minerva, Glasgow, Scotland:* Melvyn J. Percy (Nonvoting Member).

Data Center: Professor Ian Ford.

ECG Core Laboratory: Professor Peter W. Macfarlane (Coordination)

Data and Safety Monitoring Committee: *Atlanta, Georgia, USA*: Dr. William Virgil Brown (Chairman). *Essen, Germany*: Dr. Hans-Christoph Diener. *Dublin, Ireland*: Professor John Feely. *Amsterdam, The Netherlands*: Professor Pieter A. van Zwieten. *London, United Kingdom*: Dr. Stuart Pocock. *Rochester, New York, USA*: Dr. Thomas Pearson. *Glasgow, Scotland*: Professor Ian Ford (Nonvoting Member).

Endpoint Committee: *Glasgow, Scotland*: Professor Stuart M. Cobbe (Cardiology) (Chairman); Professor David J Stott (Geriatrics); Professor Peter W. Macfarlane (Cardiology [Nonvoting]). *Cork, Ireland*: Dr. Brian Sweeney (Neurology); Dr. Cillian Twomey (Geriatrics). *Leiden, The Netherlands*: Dr J. Wouter Jukema (Cardiology); Professor A. Edo Meinders (Internal Medicine and Geriatrics).

Publications Committee: *Cork, Ireland*: Professor Michael B. Murphy (Principal Investigator); Dr. Brendan M. Buckley; Dr. Michael Hyland; Professor Ivan J. Perry; Dr. Brian Sweeney; Dr Cillian Twomey. *Glasgow, Scotland*: Professor Jim Shepherd (Chairman/Principal Investigator); Professor Stuart M. Cobbe; Professor Ian Ford; Dr. Allan Gaw; Professor Peter W. Macfarlane; Professor Chris J. Packard; Professor David J. Stott. *Leiden, The Netherlands*: Dr. Gerard J. Blauw (Principal Investigator); Dr. Edward L. E. M. Bollen; Dr. J. Wouter Jukema; Dr. A. Margot Lagaay; Professor A. Edo Meinders; Dr. Rudi G. J. Westendorp.

APPENDIX B

Study end points: The primary end point of the trial is the combined end point of coronary heart disease death (definite plus suspect), nonfatal myocardial infarction (definite plus suspect), and fatal plus nonfatal stroke. When reinfarction or death occurs following a nonfatal myocardial infarction within the same period of hospitalization (up to and including 14 days from the date of admission), the subsequent event(s) will be regarded as the same event unless electrocardiographic and/or postmortem evidence suggests an infarction in a different site. When death occurs following a nonfatal stroke within a period of 28 days from the event, it will be regarded as due to a fatal stroke in the absence of other clinical events. All end points will be adjudicated by the Endpoint Committee and are defined as follows.

1. Stroke (any event that meets the criteria listed below for 1 of the following 3 categories of stroke):

(a) Ischemic stroke (1 of the following conditions must be met): (1) Rapid onset of focal neurologic deficit lasting ≥24 hours or leading to death plus evidence from neuroimaging (computed tomography or magnetic resonance imaging) showing cerebral/cerebellar infarction or no abnormality, or postmortem examination showing cerebral and/or cerebellar infarction. (2) Rapid onset of global neurologic deficit (e.g., coma) lasting ≥24 hours or leading to death plus evidence from neuroimaging showing infarction, or postmortem examination showing infarction. (3) Focal neurologic deficit (mode of onset uncertain) lasting ≥24 hours or leading to death plus evidence from neuroimaging showing infarction, or postmortem examination showing infarction.

(b) Primary intercerebral and/or cerebellar hemorrhage (1 of the following conditions must be met): (1) Rapid onset of focal neurologic deficit lasting ≥24 hours or leading to death, plus neuroimaging or postmortem examination showing primary intracerebral and/or cerebellar hemorrhage. (2) Rapid onset of global neurologic deficit (e.g., coma) lasting ≥24 hours or leading to death, plus evidence from neuroimaging or postmortem examination showing primary intracerebral and cerebellar hemorrhage. (3) Focal neurologic deficit (mode of onset uncertain) lasting ≥24 hours or leading to death, plus evidence from neuroimaging or postmortem examination showing primary intracerebral and/or cerebellar hemorrhage.

(c) Not known (1 of the following conditions must be met): (1) Rapid onset of focal neurologic deficit lasting ≥24 hours or leading to death, without neuroimaging or postmortem data available. (2) Rapid onset of global neurologic deficit (e.g., coma) lasting ≥24 hours or leading to death, without neuroimaging or postmortem data available. (3) Focal neurologic deficit (mode of onset uncertain) lasting ≥24 hours or leading to death, without neuroimaging or postmortem data available.

Note: The following conditions will be excluded from the defined end point of stroke as outlined above: (1) primary subarachnoid hemorrhage, (2) subdural or extradural hematoma, (3) traumatic intracerebral hemorrhage, (4) neurologic deficit due to major metabolic or hemodynamic disturbance, (5) venous sinus thrombosis, and (6) cerebral tumor.

2. Transient ischemic attack:

(a) Rapid onset of focal neurologic deficit or loss of monocular function lasting <24 hours.

Note: Isolated rotational vertigo, diplopia, or dysphagia are excluded from the defined end point of transient ischemic attack.

3. Definite coronary heart disease death (either or both of the following criteria must be met):

(a) Death certificate or equivalent documentation with consistent underlying or immediate cause plus: (1) Preterminal hospitalization with definite or suspect myocardial infarction; (2) previous definite angina or suspect or definite myocardial infarction when no cause other than atherosclerotic coronary heart disease death could be ascribed as the cause of death; and (3) autopsy evidence of acute coronary arterial thrombosis and/or acute myocardial infarction.

(b) Sudden and unexpected death (requires all 3 characteristics): (1) Death occurring within 1 hour after the onset of symptoms or having last been seen without them. (2) No known nonatherosclerotic acute or chronic process or event that could have been potentially lethal. (3) An "unexpected" death in a person who is not confined to the home, hospital, or other institution because of illness within 24 hours before death.

4. Definite nonfatal myocardial infarction (1 or more of the following criteria must be met):

(a) Diagnostic electrocardiogram at the time of the event.

(b) Ischemic cardiac pain (and/or unexplained acute left ventricular failure) and diagnostic enzymes.

(c) Ischemic cardiac pain and/or unexplained acute left ventricular failure with both equivocal enzymes and equivocal electrocardiogram.

(d) Diagnostic enzymes and equivocal electrocardiogram.

(e) Angiographic evidence of occlusion of a major artery with appropriate ventriculographic wall motion abnormality where previous angiogram since randomization showed no such abnormality.

(f) An electrocardiogram at an annual or at an unscheduled visit showing a myocardial infarction that was not evident on the previous electrocardiogram.

5. Suspect coronary heart disease death (either or both of the following criteria must be met):

(a) Death certificate or equivalent documentation with consistent underlying or immediate cause but neither adequate preterminal documentation of the event nor previous atherosclerotic coronary heart disease diagnosis.

(b) Rapid and unexpected death (all 3 characteristics required): (1) Death occurring between 1 and 24 hours after the onset of severe symptoms or having last been seen without them. (2) No known nonatherosclerotic acute or chronic process or event that could have been potentially lethal. (3) An "unexpected" death in a person who is not confined to the home, hospital, or other institution because of illness within 24 hours before death.

6. Suspect nonfatal myocardial infarction (any 1 of following criteria is met but the combination of (a) and (b) or (a) and (c) is not present):

(a) Ischemic cardiac pain and/or unexplained acute left ventricular failure.

Note: This definition holds only if an electrocardiogram performed during or shortly after the pain shows new equivocal changes as defined in Appendix B, or alternatively, enzymes estimated around the time of pain are equivocal.

(b) Diagnostic enzymes.

(c) Equivocal electrocardiogram and equivocal enzymes.

7. Other cardiac death: Death certificate or equivalent documentation with consistent underlying or immediate cause and adequate preterminal documentation of the event.

Note: Preterminal documentation and/or information may include hospitalization for, or diagnosis of, a cardiac-related illness other than atherosclerotic heart disease for a previous event other than the terminal event.

8. Other vascular death: Death certificate or equivalent documentation with consistent underlying or immediate cause and adequate preterminal documentation of the event.

Note: Preterminal documentation and/or information may include hospitalization for, or diagnosis of, a vascular-related illness for a previous event other than the terminal event.

9. Other death (either of the following criteria must be met):*

(a) Death certificate or equivalent documentation with diagnosis consistent with preterminal documentation and/or information.

(b) Death certificate only.

The Endpoints Committee will be responsible for the classification of all possible study end points. The Committee will receive all annual study electrocardiograms showing serial changes, information regarding domiciliary visits or hospitalizations associated with possible myocardial infarction, and information on all deaths (including postmortem reports, death certificates, hospital records, general practitioners' records, and/or interviews of family members or witnesses).

Note: Detailed definitions of diagnostic and equivocal cardiac enzyme and electrocardiographic changes are located on the PROSPER Website.

1. Kannel WB, Gordon T. Evaluation of cardiovascular risk in the elderly: the Framingham Study. *Bull N Y Acad Med* 1978;54:573–591.

2. Wenger NK. Cardiovascular disease in the elderly. *Curr Probl Cardiol* 1992;10:615–690.

3. Martyn C. Blood pressure and dementia. *Lancet* 1996;347:1130–1131.

4. Vita AJ, Terry RB, Hubert HB, Fries JF. Aging, health risks, and cumulative disability. *N Eng J Med* 1998;338:1035–1041.

5. Daviglus ML, Liu K, Greenland P, Dyer AR, Garside DB, Manheim L, Lowe LP, Rodin M, Lubitz J, Stamler J. Benefit of a favorable cardiovascular risk-factor profile in middle age with respect to Medicare costs. *N Engl J Med* 1998;339:1122–1129.

6. Folstein MF, Folstein SE, McHugh PR. Mini-mental state: a practical method for grading the cognitive state of patients for the clinician. *J Psychiatr Res* 1975;12:189–198.

7. Sigurbjornsson S, Kjartansdottir T, Johannsson M, Kristinsson J, Sigurdsson G. A pharmacokinetic evaluation of pravastatin in middle-aged and elderly volunteers. *Eur J Drug Metab Pharmacokin* 1998;23:13–18.

8. Pravastatin Multicenter Study Group. Comparative efficacy and safety of pravastatin and cholestyramine alone and combined in patients with hypercholesterolemia. *Arch Intern Med* 1993;153:1321–1329.

9. Brand N, Jolles J. Learning and retrieval rate of words presented auditorily and visually. *J Gen Psychol* 1985;112:201–210.

10. Houx PJ, Jolles J, Vreiling FW. Stroop interference: aging effects assessed with the Stroop Color-Word Test. *Experiment Aging Res* 1993;19:209–224.

11. Klein M, Ponds RWHM, Houx PJ, Jolles J. Effect of test duration on age related differences in Stroop interference. *J Clin Exptl Neuropsychol* 1997;1:66–81.

12. Smith A. The Symbol Digit Modalities Test. A neuropsychologic test for economic screening of learning and other cerebral disorders. *Learning Disorders* 1968;3:82–91.

13. Lezak MD. Verbal functions and language skills. In: Lezak MD, ed. Neuropsychological Assessment. 2nd ed. New York: Oxford University Press, 1995, 554–555.

14. Collin C, Wade DT, Davies S, Horne V. The Barthel ADL index: a reliability study. *Int Disabil Studies* 1988;10:61–63.

15. Fillenbaum GG, Smyer M. The development, validity and reliability of the OARS Multidimensional Functional Assessment Questionnaire. *J Gerontol* 1981;36:428–434.

16. Deelman BG, Brouwer WH, Zomeren AHv, Saan RJ. Functiestoornissen na trauma capitis. In: A Jennekens-Schinkel, JJ Diamant, HFA Diesfeldt, R Haaxma, eds. Neuropsychologie in Nederland Deventer. The Netherlands: Van Loghum Slaterus, 1980:253–281.

17. Rey A. L'examen psychologique dans les cas d'encephalopathie traumatique. Paris, France: Presses Universitaires de France, 1964.

18. Sacks FM, Pfeffer MA, Moye LA, Rouleau JL, Rutherford JD, Cole TG, Brown L, Warnica JW, Arnold JMO, Wun C-C, Davis BR, Braunwald E. The effect of pravastatin on coronary events after myocardial infarction in patients with average cholesterol levels. *N Engl J Med* 1996;335:1001–1009.

19. The LIPID Study Group. Prevention of cardiovascular events and death with pravastatin in patients with coronary heart disease and a broad range of initial cholesterol levels. *N Engl J Med* 1998;339:1349–1357.

20. Blauw GJ, Lagaay AM, Smelt AHM, Westendorp RGJ. Stroke, statins and cholesterol. A meta-analyses of randomized, placebo-controlled, double-blind trials with HMG-CoA-reductase inhibitors. *Stroke* 1997;28:946–950.

21. Blauw GJ, Lagaay AM, Westendorp RGJ. Statins for the prevention of stroke. *Lancet* 1998;352:144.

*When no formal written documentation is available, verbal information from relative and/or witness will be admissible and should be recorded on the appropriate study forms.
