# Supplementary material for: Pravastatin reduces all-cause mortality in elderly individuals at risk of liver fibrosis: Post hoc analysis of the PROSPER trial
Source: JHEP Rep. 2025 Feb 7;7(4):101337. doi: 10.1016/j.jhepr.2025.101337 (PMC11985108; doi:10.1016/j.jhepr.2025.101337)
Supplement: Multimedia component 5 [file mmc5.pdf]

Pravastatin reduces all-cause mortality in elderly individuals at risk of liver fibrosis: *Post hoc* analysis of the PROSPER trial

Vivian de Jong[1,2,*], Willy Theel[3], Manuel Castro Cabezas[2,3,4], Diederick E. Grobbee[1,2], Wouter Jukema[5], Stella Trompet[5,6,7]

JHEP Reports **2025**. vol. 7 | 1–6

Check for updates

Background & Aims: Metabolic dysfunction-associated steatotic liver disease (MASLD), especially the progressive stages accompanied by liver fibrosis, are associated with liver-related and cardiovascular (CV) complications in middle-aged cohorts. We evaluated whether liver fibrosis is associated with increased mortality and cause-specific endpoints in an elderly population, and whether statin treatment could reduce these risks.

Methods: PROspective Study of Pravastatin in the Elderly at Risk (PROSPER) was a double-blind randomized clinical trial comparing pravastatin to placebo in an elderly Caucasian population of 5,804 patients (>70 years of age) at increased risk of CV disease. Endpoints were composite and single (CV) endpoints and all-cause mortality. The Fibrosis-4 index (FIB-4) score was classified as: low risk of liver fibrosis (FIB-4 <2.0), indeterminate risk (2.0≤ FIB-4 ≤2.66), and high risk (FIB-4 ≥2.67). Time-to-event data were analyzed using the Cox proportional hazards model.

Results: Most participants were classified in the low FIB-4 class (n = 3,919), followed by the indeterminate (n = 1,269) and high classes (n = 561). In the placebo group, the risk of all-cause mortality increased with a high FIB-4 classification: high-class hazard ratio (HR) = 1.54 (95% CI, 1.10–2.17), compared with the low class (reference group). In the pravastatin group, the HR for all-cause mortality was not associated with FIB-4 classification: high-class HR = 1.01 (95% CI, 0.69–1.49). The interaction between FIB-4 class and treatment was significant (p = 0.049). High FIB-4 classifications were not significantly associated with major adverse cardiovascular events (MACE) or other endpoints in either arms.

Conclusions: A high FIB-4 classification is associated with increased all-cause mortality in the elderly, although pravastatin appears to mitigate this increased risk.

Clinical Trials registration: The study is registered at www.isrctn.com/(ISRCTN40976937).

© 2025 The Author(s). Published by Elsevier B.V. on behalf of European Association for the Study of the Liver (EASL). This is an open access article under the CC BY license (http://creativecommons.org/licenses/by/4.0/).

Introduction

Metabolic dysfunction-associated steatotic liver disease (MASLD) can progresses from hepatic steatosis to liver fibrosis and inflammation, and may ultimately result in liver cirrhosis, decompensation, or hepatocellular carcinoma.[1] Given the close association of MASLD with type 2 diabetes mellitus (T2DM) and obesity, its prevalence is also rapidly increasing in line with the increasing incidences of these conditions, with MASLD estimated to currently affect 38% of the global population.[2] The pathogenesis of MASLD is multifactorial, with insulin resistance as a primary driver. In cohorts of middle-aged patients with biopsy-proven MASLD/metabolic dysfunction-associated steatohepatitis (MASH), the fibrosis stage is the most important predictor of adverse liver-related outcomes and mortality.[3–5] Whether liver fibrosis is also associated with mortality in older populations remains unclear.[6]

Lately, the cardiovascular (CV) implications of MASLD have received increased attention, as reflected in updated clinical guidelines[7,8] and adoption of new nomenclature,[9] wherein MASLD is defined as hepatic steatosis in the presence of cardiometabolic risk factors. MASLD has been linked to atherosclerotic CV disease (asCVD), heart failure, and an elevated risk of CV events in middle-aged patients.[10–12] The most common causes of death in patients with MASLD appear to be related to asCVD, rather than to the liver per se.[4,7,13,14] This underscores the importance of addressing both hepatic and CV health in the management of patients with MASLD.

Recently, the first pharmacological treatment for MASLD was approved by the FDA.[15] In Europe, the only available therapies are intensive lifestyle intervention and, in selected cases, bariatric surgery.[16,17] However, the long-term success of lifestyle intervention remains low.[18] Repurposing available drugs could provide immediate support and be more cost-effective. Hydroxymethylglutaryl-coenzyme A reductase inhibitors (statins) are a class of medication primarily used to lower LDL-cholesterol levels, thereby reducing asCVD risk. It has been suggested that statins could have additional

* Corresponding author. Address: Julius Center for Health Sciences and Primary Care, Department of Global Public Health & Bioethics, University Medical Center Utrecht, Universiteitsweg 100, 3584 CX Utrecht, Utrecht, the Netherlands.
E-mail address: v.d.dejong-5@umcutrecht.nl (V. de Jong).
https://doi.org/10.1016/j.jhepr.2025.101337

anti-inflammatory and antifibrotic effects.[19–22] The current study used the PROspective Study of Pravastatin in the Elderly at Risk (PROSPER) trial data[23] to evaluate whether liver fibrosis is associated with increased mortality or cause-specific endpoints in older patients at elevated CV risk. We also investigated whether pravastatin treatment can reduce any increased risks.

Patients and methods

Study population

PROSPER was a randomized double-blind clinical trial investigating the effect of pravastatin in an older population with a history or increased risk of CVD (*i.e.* because of smoking, hypertension, or diabetes). The detailed study set-up has been published previously.[23,24] Between 1997 and 1999, 5,804 people aged 70–82 years from Scotland, Ireland, and the Netherlands were included and randomized in a 1:1 ratio to receive either 40 mg pravastatin or matching placebo. The mean follow-up duration was 3.2 years and the study was completed in 2002.

Endpoints

The composite endpoint of the trial was major adverse CV events (MACE), defined as definite or suspected coronary heart disease death, nonfatal myocardial infarction (MI), and either fatal or nonfatal stroke. The other endpoints included in this analysis were: fatal/nonfatal stroke and transient ischaemic attack (TIA); fatal/nonfatal MI; heart failure; all-cause mortality; cancer mortality; and CV mortality.

FIB-4 score

The presence of liver fibrosis was estimated using the Fibrosis-4 Index (FIB-4)[25] The FIB-4 algorithm is based on age, aspartate aminotransferase (AST), alanine aminotransferase (ALT), and platelet count, and was calculated at baseline using Equation 1:

$$\text{FIB-4} = [\text{age (years)} \times \text{AST (U/L)}] \Big/ \left[\text{platelet count} \left(10^9/\text{L}\right) \times \sqrt{ALT}\left(U/L\right)\right] \quad [1]$$

Given that the population was above 65 years of age, we used the stricter age-corrected lower cut-off value (2.0 instead of 1.3) as proposed by McPherson *et al.*[26] The population was split into three classes based on the following cut-off values: ruled-out advanced fibrosis (FIB-4 <2.0), indeterminate results (2.0 ≤ FIB-4 ≤2.66), and rule-in advanced fibrosis (FIB-4 ≥2.67). Reports from the LITMUS and LiverScreen consortia indicate that FIB-4 has adequate prognostic abilities at the population level.[27,28]

Statistical analysis

Analyses were based on the intention-to-treat dataset. Population descriptive statistics were provided stratified by FIB-4 classification. Continuous data are presented as mean ± SE, and categorical data as count and proportion. The time-to-event data were analyzed with Cox proportional hazards models according to FIB-4 classification, for the placebo and pravastatin groups separately. For each endpoint, either time to first occurrence of the event or study closure (censored observation) was taken, depending on which came first. The models were adjusted for sex, current smokers, BMI, diabetes, and history of CVD. For each endpoint, the hazard ratio (HR) and its 95% CI were estimated. Data analysis was conducted using SPSS (v. 29).

Results

The baseline FIB-4 score was calculated for 5,749 participants of the total intention-to-treat population of 5,804 participants, given that the values used to calculate FIB-4 were missing for 55 participants (Fig. S1). The mean (±SE) FIB-4 in the placebo group and treatment group was similar (1.80 ± 0.01 placebo *vs.* 1.81 ± 0.01 treatment). Most participants were classified in the low FIB-4 class (n = 3,919), followed by the indeterminate class (n = 1,269), with fewest in the high FIB-4 class (n = 561). In the lowest FIB-4 class, most participants were female, whereas, in the indeterminate and high FIB-4 classes, most were male. The mean age (±SE) of the classes ranged from 74.9 (±0.07) to 76.4 (±0.21) years. Around 9–13% of the population had diabetes. A slightly increasing prevalence of a history of CVD was seen from the low (42%), to the indeterminate (47%) to the high FIB-4 (53%) classes (p <0.001). In terms of medication, use of metformin and insulin was minimal and did not differ between the classes. Use of angiotensin-converting enzyme (ACE)-/angiotensin II-inhibitors did not differ significantly between the classes or between the arms. The baseline characteristics are summarized in Table 1.

A high FIB-4 classification was not significantly associated with the composite endpoint MACE (Table 2). In terms of the single endpoints, the risk of all-cause mortality was increased with higher FIB-4 classification in the placebo group (Fig. 1). In the placebo group, the high FIB-4 class had a HR of 1.54 (95% CI, 1.10–2.17) for all-cause mortality compared with the low FIB-4 class (reference group). However, in the pravastatin group, the HRs did not increase with a higher FIB-4 classification. In the pravastatin group, the high FIB-4 class had a HR of 1.01 (95% CI, 0.69–1.49) for all-cause mortality compared with the low FIB-4 class (reference group). The interaction term between FIB-4 class and statin treatment was significant (p = 0.049), indicating that the effect of treatment depended on FIB-4 classification (or vice versa). A higher FIB-4 classification was not associated with any of the other single endpoints studied: fatal/non-fatal stroke/TIA; fatal/nonfatal MI; heart failure; or cancer mortality (Table 2). A similar table showing only subjects that completed per protocol analysis is shown in Supplemental Material, Table S1.

Discussion

In the current analysis of the PROSPER trial data, a high FIB-4 classification was associated with increased all-cause mortality in older patients in the placebo arm. However, pravastatin treatment appears to abolish this risk.

The most studied prognostic outcome in relation to FIB-4 is all-cause mortality. Systematic reviews and meta-analyses indicate that FIB-4 can accurately predict all-cause mortality.[27,29] A individual participant data meta-analysis showed that FIB-4 is as good as histological fibrosis staging to predict a composite endpoint of all-cause mortality and liver-related outcomes (mean age 54 years).[30] In our older population, a

Table 1. Baseline characteristics of the PROSPER population stratified by FIB-4 classification and treatment.

	Placebo				Pravastatin			
	**Low FIB-4**	**Indeterminate FIB-4**	**High FIB-4**	***p* value**	**Low FIB-4**	**Indeterminate FIB-4**	**High FIB-4**	***p* value**
Participants (n)	1,975	636	273		1,944	633	288	
FIB-4	1.45 (0.01)	2.27 (0.01)	3.23 (0.04)	NA	1.45 (0.01)	2.28 (0.01)	3.18 (0.03)	NA
Age (years)	74.9 (0.07)	76.0 (0.13)	76.4 (0.21)	NA	75.1 (0.07)	75.9 (0.13)	76.0 (0.20)	NA
Females (n, %)	1,115 (56.5)	271 (42.6)	106 (38.8)	<0.001	1,088 (56.0)	286 (45.2)	105 (36.5)	<0.001
BMI (kg/m^2)	26.9 (0.10)	26.7 (0.16)	26.2 (0.27)	0.007	27.0 (0.09)	26.6 (0.16)	26.1 (0.24)	<0.001
Current smokers (n, %)	581 (29.3)	146 (23.0)	70 (25.6)	0.010	540 (27.8)	151 (23.9)	58 (20.1)	0.002
Systolic blood pressure (mmHg)	154 (0.5)	156 (0.9)	156 (1.3)	0.081	154 (0.5)	155 (0.9)	156 (1.4)	0.203
**(Fasting) clinical values**								
Glucose (mmol/L)	5.48 (0.03)	5.44 (0.06)	5.49 (0.10)	0.771	5.49 (0.03)	5.35 (0.05)	5.39 (0.09)	0.061
Insulin (mIU/L)	10.6 (0.26)	9.6 (0.40)	9.8 (0.46)	0.070	10.5 (0.24)	10.1 (0.43)	10.5 (0.73)	0.660
HOMA-IR	2.64 (0.07)	2.45 (0.13)	2.53 (0.15)	0.262	2.64 (0.07)	2.47 (0.11)	2.59 (0.19)	0.409
Triglycerides (mmol/L)	1.57 (0.02)	1.50 (0.03)	1.35 (0.04)	<0.001	1.58 (0.02)	1.52 (0.03)	1.38 (0.04)	<0.001
HDL-cholesterol (mmol/L)	1.27 (0.01)	1.28 (0.01)	1.31 (0.02)	0.112	1.28 (0.01)	1.29 (0.02)	1.32 (0.02)	0.163
LDL-cholesterol (mmol/L)	3.82 (0.02)	3.73 (0.03)	3.63 (0.05)	<0.001	3.82 (0.02)	3.76 (0.03)	3.73 (0.05)	0.029
Creatinine (µmol/L)	100.3 (0.50)	102.8 (0.91)	101.3 (1.24)	0.067	101.0 (0.51)	102.1 (0.89)	102.5 (1.23)	0.165
AST (U/L)	22.9 (0.16)	27.9 (0.31)	33.4 (0.86)	NA	22.6 (0.16)	27.6 (0.33)	34.6 (0.80)	NA
ALT (U/L)	23.6 (0.24	23.3 (0.47)	23.7 (1.03)	NA	23.0 (0.22)	22.6 (0.45)	26.2 (1.08)	NA
Platelets (10^9/L)	253.8 (1.22)	199.6 (1.41)	171.4 (2.02)	NA	254.4 (1.33)	199.1 (1.44)	170.0 (1.97)	NA
**Medical history**								
Diabetes (n, %)	217 (11.0)	64 (10.1)	34 (12.5)	0.792	210 (10.8)	56 (8.8)	31 (10.8)	0.501
Vascular disease (n, %)	811 (41.1)	291 (45.8)	146 (53.5)	<0.001	841 (43.3)	302 (47.7)	150 (52.1)	0.002
**Medication use**								
ACE inhibitors/angiotensin II inhibitors (n, %)	334 (16.9)	123 (19.3)	56 (20.5)	0.067	357 (18.4)	117 (18.5)	69 (24.0)	0.065
Metformin (n, %)	125 (6.3)	35 (5.5)	20 (7.3)	0.886	125 (6.4)	28 (4.4)	19 (6.6)	0.438
Insulin (n, %)	22 (1.1)	6 (0.9)	2 (0.7)	0.521	14 (0.7)	4 (0.6)	3 (1.0)	0.709

Continuous data are presented as mean (±SE), categorical data as count (%). FIB-4 classes: low FIB-4 <2.0; indeterminate FIB-4, 2.0–2.66; high FIB-4 ≥2.67. Numerical variables compared using ANOVA, categorical using Chi-square tests; *p* <0.05 were considered statistically significant (indicated in bold).

NA, testing not applicable, because these variables are part of the FIB-4 algorithm. ACE, angiotensin-converting enzyme; ALT, alanine aminotransferase; AST, aspartate aminotransferase; FIB-4, Fibrosis-4 index; HOMA-IR, Homeostatic model assessment for insulin resistance.

Table 2. Hazard ratios and corresponding 95% CI for each endpoint for each FIB-4 class in the placebo arm and the treatment arm.

	Placebo (HR and 95% CI)			Pravastatin (HR and 95% CI)		
**FIB-4 class**	**Low (n = 1,975)**	**Indeterminate (n = 636)**	**High (n = 273)**	**Low (n = 1,944)**	**Indeterminate (n = 633)**	**High (n = 288)**
Major adverse cardiovascular events	1.0	0.98 (0.79–1.22)	0.82 (0.59–1.14)	1.0	1.03 (0.81–1.31)	0.98 (0.70–1.37)
**Single endpoints**						
Fatal/nonfatal stroke/transient ischemic attack	1.0	1.20 (0.79–1.81)	1.15 (0.65–2.04)	1.0	0.98 (0.64–1.52)	1.34 (0.79–2.26)
Fatal/nonfatal myocardial infarction	1.0	0.92 (0.71–1.19)	0.72 (0.48–1.06)	1.0	1.04 (0.78–1.38)	0.90 (0.60–1.36)
Heart failure	1.0	0.99 (0.64–1.52)	0.77 (0.40–1.49)	1.0	0.89 (0.55–1.44)	1.26 (0.71–2.24)
All-cause mortality	1.0	1.28 (0.97–1.68)	1.54 (1.10–2.17)*	1.0	0.97 (0.73–1.29)	1.01 (0.69–1.49)
Cancer incidence	1.0	1.12 (0.80–1.57)	1.14 (0.72–1.82)	1.0	0.87 (0.63–1.20)	0.93 (0.60–1.43)
Cancer mortality	1.0	1.16 (0.70–1.91)	1.15 (0.57–2.33)	1.0	0.85 (0.53–1.36)	0.95 (0.50–1.79)
Cardiovascular mortality	1.0	1.39 (0.97–2.00)	1.11 (0.65–1.89)	1.0	1.08 (0.71–1.64)	1.15 (0.66–2.00)

Low FIB-4 is the reference class (HR set to 1). The Cox proportional hazards models were adjusted for sex, smoking, BMI, diabetes, and history of vascular disease. *95% CI corresponding to alpha = 0.05, does not overlap with 1. FIB-4, Fibrosis-4 index; HR, hazard ratio.

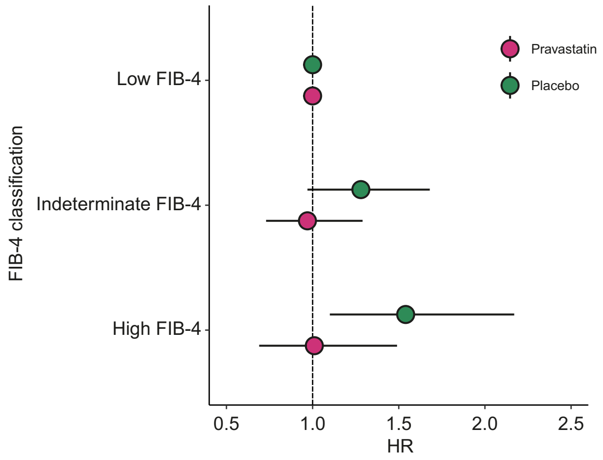

Fig. 1. Hazard ratio (dots) and 95% CI (bars) for all-cause mortality for each FIB-4 classification compared to the reference class of low FIB-4 (HR set at 1) for the placebo and treatment group. The Cox proportional hazards model was adjusted for sex, smoking, BMI, diabetes, and history of vascular disease. *95% CI corresponding to alpha = 0.05, does not overlap with 1. FIB-4, Fibrosis-4 index; HR, hazard ratio.

high FIB-4 classification (≥2.67) was also associated with an increased risk of all-cause mortality in the placebo arm. By contrast, an older general population cohort showed that neither liver steatosis nor liver fibrosis were associated with increased mortality (mean age 74 years, median follow-up almost 7 years).[31] Whereas age and BMI were relatively similar between both studies, the prevalence of diabetes was higher in the general population cohort, whereas the presence of CVD was higher in our study. In addition, the assessment of fibrosis differed between the studies (FIB-4 *vs.* transient elastography). A registry-based study of Taiwanese patients with heart failure (mean age 75 years) also reported that FIB-4 was predictive of mortality in older people,[32] although different FIB-4 cut-offs were used compared with the current analysis.

Several studies describe the prognostic relation between FIB-4 and CVD, although the results are less clear than for mortality. In a large meta-analysis, FIB-4 was not associated with CV events, although the number of events was relatively low (78 events in n = 2,518; 3%).[30] By contrast, when using electronic health record data of patients with obesity and T2DM (6,002 CV events in n = 44,481; 13%) a higher FIB-4 classification was predictive of CV events (mean age 59 years).[33] In the NHANES database, a high FIB-4 (≥2.67) was also predictive of CV events (mean age 53 years).[34] In our older population, we did not find any association with MACE or any of the other CV endpoints. The power of our post hoc analysis might have been too low to detect differences in other single endpoints, although a relatively high number of combined CV events was observed for MACE (870 events in n = 5,749; 15%). However, the prospective relationships between FIB-4 and all-cause mortality might be stronger than for combined and single CV endpoints.

Statins are well known for their cholesterol-lowering effects. Lowering LDL-cholesterol might protect patients who are at increased risk of CV complications because of liver fibrosis, regardless of any potential effect on the fibrosis itself. Statins were associated with a decrease in mortality in patients with MASLD in the NHANES database.[22] Our study confirms these

retrospective NHANES findings, because we show that treatment with statins decreased all-cause mortality in PROSPER.

Statins might have additional anti-inflammatory and anti-fibrotic effects.[35] Preclinical data demonstrated a beneficial effect of statins on diet-induced MASH[36,37] and fibrosis.[37] Only two small-sized randomized controlled trials on statins in MASLD have been performed,[38,39] which were both classified as having a high risk of bias in the only Cochrane review to-date,[40] and, therefore, no definite conclusion was reached. Given that no large-scale trials with adequate follow-up are available, the clinical evidence of statins in MASLD is limited to cohort data and post hoc analyses of RCTs. Statins were associated with protection against MASH and fibrosis in a cross-sectional study.[41] Recently, two large meta-analyses reported that statin use was associated with a lower risk of MASH and fibrosis.[19,20] Although statins are considered safe in patients with MASLD,[42] older patients with liver fibrosis might be at a slightly increased risk of liver toxicity. In the current analysis, potential adverse effects did not compensate for the survival benefit. Nevertheless, in older populations, close monitoring of those with pre-existing liver conditions, multimorbidity, and polypharmacy remains important.

The prevalence of MASLD is higher in patients with T2DM compared with the general population,[43] although this might be mainly driven by hepatic steatosis and insulin resistance. In PROSPER, the prevalence of T2DM was 9–13% and did not differ within the different fibrosis classifications.

To appreciate these findings, some aspects of this study need to be addressed. The strength of the current study is the use of a large dataset from a randomized controlled trial with an adequate follow-up time (4 years). The older study population at risk of CVD is comparable to patients who would be considered for statin treatment in practice. The main limitation of this study was the lack of direct liver assessment by either imaging or biopsy, and FIB-4 was selected as proxy for liver fibrosis. FIB-4 has been validated as a diagnostic marker for advanced fibrosis at the population level, as reflected in available guidelines.[44] However, the diagnostic accuracy of FIB-4 in specific subpopulations, such as patients with T2DM, is lower. The accuracy of its use as prognostic marker is also less studied, although recent data show that FIB-4 has discriminative power in patients with T2DM.[33] In addition, FIB-4 is mostly validated (as a diagnostic marker) in populations under 65 years of age. The FIB-4 algorithm includes age and, thus, likely overestimates the presence of fibrosis in older participants. Therefore, in this analysis, we used age-specific cut-offs as proposed by McPherson *et al.*[26] and adopted in the 2024 EASL–EASD–EASO Clinical Practice Guidelines.[8] PROSPER compared pravastatin with placebo, while other types of statin (i.e., simvastatin and atorvastatin) are more commonly prescribed. Given that the mechanism of action of statins is similar, we expect the results to also be generalizable to other statins. A high level of high intraindividual variation was observed in this study, possibly because the response to statin treatment might differ based on underlying genotype or metabolic profile.[41]

Non-randomized studies have indicated potential benefits of statin use in middle-aged patients with MASLD,[19,22,41] but no consensus or standard recommendation has been reached. Our analysis of PROSPER suggests that the beneficial effects of statins also extend to an older population with a high risk of liver fibrosis.

In conclusion, our findings obtained in a large population of older participants at elevated CV risk support the view that increased risks of fibrosis lead to increased risks of all-cause mortality. Moreover, the results of a randomized comparison suggest that pravastatin treatment, or possibly statin treatment in general, mitigates these risks.

Affiliations

[1]*Julius Center for Health Sciences and Primary Care, University Medical Center Utrecht, Utrecht, the Netherlands;* [2]*Julius Clinical, Zeist, the Netherlands;* [3]*Department of Internal Medicine, Franciscus Gasthuis & Vlietland, Rotterdam, the Netherlands;* [4]*Department of Endocrinology, Erasmus MC Medical Center, Rotterdam, the Netherlands;* [5]*Department of Cardiology, Leiden University Medical Centre, Leiden, the Netherlands;* [6]*Netherlands Heart Institute, Utrecht, the Netherlands;* [7]*Department of Internal Medicine, Section Gerontology and Geriatrics, Leiden University Medical Centre, Leiden, the Netherlands*

Abbreviations

ACE, angiotensin-converting enzyme; ALT, alanine aminotransferase; asCVD, atherosclerotic CVD; AST, aspartate aminotransferase; CV, cardiovascular; CVD, cardiovascular disease; FIB-4, Fibrosis-4; HOMA-IR, Homeostatic Model Assessment for Insulin Resistance; HR, hazard ratio; MACE, major adverse cardiovascular events; MASH, metabolic dysfunction-associated steatohepatitis; MASLD, Metabolic dysfunction-associated steatotic liver disease; MI, myocardial infarction; PROSPER, PROspective Study of Pravastatin in the Elderly at Risk; T2DM, type 2 diabetes mellitus; TIA, transient ischemic attack.

Financial support

The authors received no specific funding for this work. The original PROSPER study was supported by an investigator-initiated grant obtained from Bristol-Myers Squibb, USA. Prof. Dr. J. W. Jukema is an Established Clinical Investigator of the Netherlands Heart Foundation (grant 2001 D 032). The funders had no role in study design, data collection and analysis, decision to publish, or preparation of the manuscript.

Conflicts of interest

The authors have declared no competing interests.

Please refer to the accompanying ICMJE disclosure forms for further details.

Authors' contributions

Study concept and design; MCC, ST, VDJ. Statistical analysis; ST. Interpretation of data; ST, VDJ, MCC, DG. Drafting of the manuscript; VDJ. Critical revision of the manuscript; MCC, ST, DG, WT, WJ. Supervision: MCC, DG.

Data availability statement

Data cannot be shared publicly due to ethical constraints, but can be requested from the PROSPER Scientific Committee (vice chairman of the PROSPER Scientific Committee: Naveed Sattar, naveed.sattar@glasgow.ac.uk) or from the co-author and PROSPER PI J.W. Jukema (j.w.jukema@lumc.nl).

Acknowledgements

The authors thank the PROSPER study team and the patients who participated in the study.

Supplementary data

Supplementary data to this article can be found online at https://doi.org/10.1016/j.jhepr.2025.101337.

References

Author names in bold designate shared co-first authorship

[1] Loomba R, Friedman SL, Shulman GI. Mechanisms and disease consequences of nonalcoholic fatty liver disease. Cell 2021;184:2537–2564.
[2] Younossi ZM, Golabi P, Paik JM, et al. The global epidemiology of nonalcoholic fatty liver disease (NAFLD) and nonalcoholic steatohepatitis (NASH): a systematic review. Hepatology 2023;77:1335–1347.
[3] Sanyal AJ, Van Natta ML, Clark J, et al. Prospective study of outcomes in adults with nonalcoholic fatty liver disease. N Engl J Med 2021;385:1559–1569.
[4] Ekstedt M, Hagström H, Nasr P, et al. Fibrosis stage is the strongest predictor for disease-specific mortality in NAFLD after up to 33 years of follow-up. Hepatology 2015;61:1547–1554.
[5] Taylor RS, Taylor RJ, Bayliss S, et al. Association between fibrosis stage and outcomes of patients with nonalcoholic fatty liver disease: a systematic review and meta-analysis. Gastroenterology 2020;158:1611–1625.
[6] Bilson J, Byrne CD. Fatty liver disease and risk of all cause and cause-specific mortality outcomes in the older population. Hepatobiliary Surg Nutr 2023;12:949.
[7] Duell PB, Welty FK, Miller M, et al. Nonalcoholic fatty liver disease and cardiovascular risk: a scientific statement from the American Heart Association. Arterioscler Thromb Vasc Biol 2022;42:e168–e185.
[8] European Association for the Study of Liver. EASL–EASD–EASO Clinical Practice Guidelines on the management of metabolic dysfunction-associated steatotic liver disease (MASLD). J Hepatol 2024;81:492–542.
[9] Rinella ME, Lazarus JV, Ratziu V, et al. A multi-society Delphi consensus statement on new fatty liver disease nomenclature. Hepatology 2023;78:1966–1986.
[10] Mantovani A, Csermely A, Petracca G, et al. Non-alcoholic fatty liver disease and risk of fatal and non-fatal cardiovascular events: an updated systematic review and meta-analysis. Lancet Gastroenterol Hepatol 2021;6:903–913.
[11] Baratta F, Pastori D, Angelico F, et al. Nonalcoholic fatty liver disease and fibrosis associated with increased risk of cardiovascular events in a prospective study. Clin Gastroenterol Hepatol 2020;18:2324–2331.
[12] Driessen S, Francque SM, Anker SD, et al. Metabolic dysfunction associated steatotic liver disease and the heart. Hepatology 2023. https://doi.org/10.1097/HEP.0000000000000735.
[13] Byrne CD, Targher G. NAFLD: a multisystem disease. J Hepatol 2015;62(1 Suppl):S47–S64.
[14] Targher G, Byrne CD, Lonardo A, et al. Non-alcoholic fatty liver disease and risk of incident cardiovascular disease: a meta-analysis. J Hepatol 2016;65:589–600.
[15] Harrison SA, Bedossa P, Guy CD, et al. A Phase 3, randomized, controlled trial of resmetirom in NASH with liver fibrosis. N Engl J Med 2024;390:497–509.
[16] Marchesini G, Day CP, Dufour JF, et al. EASL–EASD–EASO Clinical Practice Guidelines for the management of non-alcoholic fatty liver disease. J Hepatol 2016;64:1388–1402.
[17] Ratziu V. Non-pharmacological interventions in non-alcoholic fatty liver disease patients. Liver Int 2017;37:90–96.
[18] Haigh L, Kirk C, El Gendy K, et al. The effectiveness and acceptability of Mediterranean diet and calorie restriction in non-alcoholic fatty liver disease (NAFLD): a systematic review and meta-analysis. Clin Nutr 2022;41:1913–1931.
[19] **Ayada I**, **van Kleef LA**, Zhang H, et al. Dissecting the multifaceted impact of statin use on fatty liver disease: a multidimensional study. EBioMedicine 2022;87:104392.
[20] Fatima K, Moeed A, Waqar E, et al. Efficacy of statins in treatment and development of non-alcoholic fatty liver disease and steatohepatitis: a systematic review and meta-analysis. Clin Res Hepatol Gastroenterol 2022;46:101816.
[21] Dai W, Xu B, Li P, et al. Statins for the treatment of nonalcoholic fatty liver disease and nonalcoholic steatohepatitis: a systematic review and meta-analysis. Am J Ther 2023;30:e17–e25.
[22] Ng CH, Teng ML, Chew NW, et al. Statins decrease overall mortality and cancer related mortality but are underutilized in NAFLD: a longitudinal analysis of 12,538 individuals. Expert Rev Gastroenterol Hepatol 2022;16:895–901.
[23] Shepherd J, Blauw GJ, Murphy MB, et al. The design of a prospective study of pravastatin in the elderly at risk (PROSPER). Am J Cardiol 1999;84:1192–1197.
[24] Shepherd J, Blauw GJ, Murphy MB, et al. Pravastatin in elderly individuals at risk of vascular disease (PROSPER): a randomised controlled trial. Lancet 2002;360:1623–1630.
[25] Sterling RK, Lissen E, Clumeck N, et al. Development of a simple noninvasive index to predict significant fibrosis in patients with HIV/HCV coinfection. Hepatology 2006;43:1317–1325.
[26] McPherson S, Hardy T, Dufour JF, et al. Age as a confounding factor for the accurate non-invasive diagnosis of advanced NAFLD fibrosis. Am J Gastroenterol 2017;112:740.
[27] Lee J, Vali Y, Boursier J, et al. Prognostic accuracy of FIB-4, NAFLD fibrosis score and APRI for NAFLD-related events: a systematic review. Liver Int 2021;41:261–270.
[28] Serra-Burriel M, Juanola A, Serra-Burriel F, et al. Development, validation, and prognostic evaluation of a risk score for long-term liver-related outcomes in the general population: a multicohort study. Lancet 2023;402:988–996.
[29] Cianci N, Subhani M, Hill T, et al. Prognostic non-invasive biomarkers for all-cause mortality in non-alcoholic fatty liver disease: a systematic review and meta-analysis. World J Hepatol 2022;14:1025–1037.
[30] Mózes FE, Lee JA, Vali Y, et al. Performance of non-invasive tests and histology for the prediction of clinical outcomes in patients with non-alcoholic fatty liver disease: an individual participant data meta-analysis. Lancet Gastroenterol Hepatol 2023;8:704–713.
[31] Van Kleef LA, Sonneveld MJ, Kavousi M, et al. Fatty liver disease is not associated with increased mortality in the elderly: a prospective cohort study. Hepatology 2023;77:585.
[32] Tseng CH, Huang WM, Yu WC, et al. The fibrosis-4 score is associated with long-term mortality in different phenotypes of acute heart failure. Eur J Clin Invest 2022;52:e13856.
[33] Anstee QM, Berentzen TL, Nitze LM, et al. Prognostic utility of Fibrosis-4 Index for risk of subsequent liver and cardiovascular events, and all-cause mortality in individuals with obesity and/or type 2 diabetes: a longitudinal cohort study. Lancet Reg Health Eur 2024;36:100780.
[34] Chew NWS, Ng CH, Chan KE, et al. FIB-4 predicts MACE and mortality in cardiovascular mortality in patients with non-alcoholic fatty liver disease. Can J Cardiol 2022;38:1779–1780.
[35] Schierwagen R, Uschner FE, Magdaleno F, et al. Rationale for the use of statins in liver disease. Am J Physiol Gastrointest Liver Physiol 2017;312:G407–G412.
[36] Bravo M, Raurell I, Hide D, et al. Restoration of liver sinusoidal cell phenotypes by statins improves portal hypertension and histology in rats with NASH. Sci Rep 2019;9:1–12.
[37] Inia JA, Stokman G, Pieterman EJ, et al. Atorvastatin attenuates diet-induced non-alcoholic steatohepatitis in APOE*3-Leiden mice by reducing hepatic inflammation. Int J Mol Sci 2023;24:7818.
[38] Athyros VG, Mikhailidis DP, Didangelos TP, et al. Effect of multifactorial treatment on non-alcoholic fatty liver disease in metabolic syndrome: a randomised study. Curr Med Res Opin 2006;22:873–883.
[39] Nelson A, Torres DM, Morgan AE, et al. A pilot study using simvastatin in the treatment of nonalcoholic steatohepatitis: a randomized placebo-controlled trial. J Clin Gastroenterol 2009;43:990–994.
[40] Eslami L, Merat S, Malekzadeh R, et al. Statins for non-alcoholic fatty liver disease and non-alcoholic steatohepatitis. Cochrane Database Syst Rev 2013;2013:CD008623.
[41] Dongiovanni P, Petta S, Mannisto V, et al. Statin use and non-alcoholic steatohepatitis in at risk individuals. J Hepatol 2015;63:705–712.
[42] Pastori D, Pani A, Di Rocco A, et al. Statin liver safety in non-alcoholic fatty liver disease: a systematic review and metanalysis. Br J Clin Pharmacol 2022;88:441–451.
[43] Younossi ZM, Golabi P, de Avila L, et al. The global epidemiology of NAFLD and NASH in patients with type 2 diabetes: a systematic review and meta-analysis. J Hepatol 2019;71:793–801.
[44] European Association for the Study of the Liver. EASL Clinical Practice Guidelines on non-invasive tests for evaluation of liver disease severity and prognosis - 2021 update. J Hepatol 2021;75:659–689.

Keywords: MASLD; FIB-4; Statin; Advanced fibrosis.
Received 30 July 2024; received in revised form 20 January 2025; accepted 22 January 2025; Available online 7 February 2025

Journal of Hepatology, Volume *7*

Supplemental information

Pravastatin reduces all-cause mortality in elderly individuals at risk of liver fibrosis: *Post hoc* analysis of the PROSPER trial

Vivian de Jong, Willy Theel, Manuel Castro Cabezas, Diederick E. Grobbee, Wouter Jukema, and Stella Trompet

Pravastatin reduces all-cause mortality in elderly individuals at risk of liver fibrosis: *Post hoc* analysis of the PROSPER trial

Vivian Desiree de Jong, Willy Theel, Manuel Castro Cabezas, Diederick Grobbee, Wouter Jukema, Stella Trompet

Table of contents

Fig. S1……………………………………………………………………………….2

Table S1……………………………………………………………………………..3

Fig. S1: patient flow through the original trial, with addition of missing FIB-4. **The** ITT population was n=2,913 for placebo and n=2,891 for treatment. In the placebo for n=29 values to calculate FIB-4 were missing, in the treatment group for n=26 values to calculate FIB-4 were missing. Resulting in a total population of n=2,884 in placebo and n=2,865 for treatment for this post-hoc analysis.

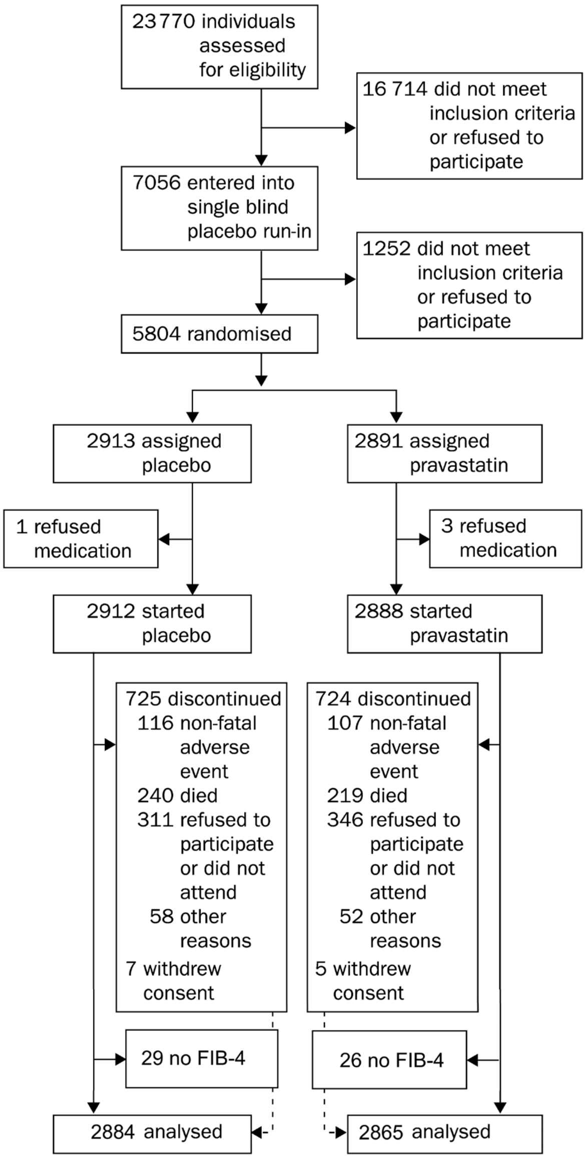

Table S1: Hazard ratios (HR) and corresponding 95% CI for each endpoint for each FIB-4 class in the placebo arm and the treatment arm. Low FIB-4 is the reference class (HR set to 1). The Cox proportional hazards models were adjusted for sex, smoking, BMI, diabetes and history of vascular disease. ONLY in subjects that completed the per protocol analysis (people that withdraw consent were left out). *95% confidence interval corresponding to alpha=0.05, does not overlap with 1.

	**Placebo**			**Pravastatin**		
**FIB-4 class**	Low<br>N=1681	Indeterminate<br>N=549	High<br>N=244	Low<br>N=1640	Indeterminate<br>N=553	High<br>N=253
**MACE**	1.0	0.98<br>(0.77-1.26)	0.89<br>(0.63-1.26)	1.0	1.00<br>(0.77-1.30)	0.97<br>(0.67-1.40)
**Single endpoints**						
**Fatal/non-fatal Stroke/TIA**	1.0	1.28<br>(0.79-2.05)	1.42<br>(0.77-2.60)	1.0	0.81<br>(0.48-1.37)	1.34<br>(0.75-2.40)
**Fatal/nonfatal MI**	1.0	0.90<br>(0.68-1.19)	0.76<br>(0.50-1.14)	1.0	1.07<br>(0.79-1.44)	0.91<br>(0.59-1.41)
**Heart failure**	1.0	0.88<br>(0.54-1.44)	0.80<br>(0.40-1.61)	1.0	0.96<br>(0.57-1.61)	1.42<br>(0.77-2.61)
**All-cause mortality**	1.0	1.26<br>(0.96-1.65)	1.47<br>**(1.05-2.07)***	1.0	0.94<br>(0.71-1.25)	0.97<br>(0.66-1.43)
**Cancer Incidence**	1.0	1.24<br>(0.87-1.77)	1.19<br>(0.73-1.94)	1.0	0.83<br>(0.58-1.18)	0.94<br>(0.59-1.49)
**Cancer mortality**	1.0	1.31<br>(0.69-1.87)	1.08<br>(0.54-2.19)	1.0	0.81<br>(0.51-1.31)	0.91<br>(0.48-1.72)
**CV mortality**	1.0	1.38<br>(0.96-1.98)	1.07<br>(0.63-1.82)	1.0	1.38<br>(0.96-1.98)	1.07<br>(0.63-1.98)
